# Pivotal roles of biglycan and decorin in regulating bone mass, water retention, and bone toughness

**DOI:** 10.1038/s41413-024-00380-2

**Published:** 2025-01-02

**Authors:** Rui Hua, Yan Han, Qingwen Ni, Roberto J. Fajardo, Renato V. Iozzo, Rafay Ahmed, Jeffry S. Nyman, Xiaodu Wang, Jean X. Jiang

**Affiliations:** 1 Department of Biochemistry and Structural Biology, University of Texas Health Science Center, San Antonio, TX USA; 2 Department of Mechanical Engineering, University of Texas at San Antonio, San Antonio, TX USA; 3 Department of Physics, Texas A&M International University, Laredo, TX USA; 4 School of Osteopathic Medicine, University of the Incarnate Word, San Antonio, TX USA; 5 Department of Pathology & Genomic Medicine, Sidney Kimmel Medical Collage, Thomas Jefferson University, Philadelphia, PA USA; 6 Department of Orthopaedic Surgery, Vanderbilt University Medical Center, Nashville, TN USA; 7 United States Department of Veterans Affairs, Tennessee Valley Healthcare System, Nashville, TN USA

**Keywords:** Bone quality and biomechanics, Bone, Pathogenesis

## Abstract

Proteoglycans, key components of non-collagenous proteins in the bone matrix, attract water through their negatively charged glycosaminoglycan chains. Among these proteoglycans, biglycan (Bgn) and decorin (Dcn) are major subtypes, yet their distinct roles in bone remain largely elusive. In this study, we utilized single knockout (KO) mouse models and successfully generated double KO (dKO) models despite challenges with low yield. *Bgn* deficiency, but not *Dcn* deficiency, decreased trabecular bone mass, with more pronounced bone loss in dKO mice. Low-field nuclear magnetic resonance measurements showed a marked decrease in bound water among all KO groups, especially in *Bgn* KO and dKO mice. Moreover, both *Bgn* KO and dKO mice exhibited reduced fracture toughness compared to *Dcn* KO mice. Dcn was significantly upregulated in *Bgn* KO mice, while a modest upregulation of Bgn was observed in *Dcn* KO mice, indicating Bgn’s predominant role in bone. High resolution atomic force microscopy showed decreased in situ permanent energy dissipation and increased elastic modulus in the extrafibrillar matrix of *Bgn*/*Dcn* deficient mice, which were diminished upon dehydration. Furthermore, we found that both Bgn and Dcn are indispensable for the activation of ERK and p38 MAPK signaling pathways. Collectively, our results highlight the distinct and indispensable roles of Bgn and Dcn in maintaining bone structure, water retention, and bulk/in situ tissue properties in the bone matrix, with Bgn exerting a predominant influence.

## Introduction

Bone has a highly hierarchical structure that is comprised of mineralized collagen fibrils embedded in the extrafibrillar matrix. By mass, its composition includes approximately 60% hard, mineral phase (primarily semi-crystalline, carbonated hydroxyapatite), 30% soft, organic phase involving type I collagen fibrils and non-collagenous proteins, and 10% water.^1^ Consequently, the biomechanical characteristics of bone are influenced by the quality and spatial organization of these components. The contributions of mineral and collagen phases to bone mechanical competence have been well documented.^2–6^ However, it was not until recently that a substantial role for the non-collagenous proteins has gained attention. There are structural proteins dispersed throughout the bone extracellular matrix, which have been shown to be directly involved in bone deformation and failure.^7,8^ As a major part of non-collagenous proteins, proteoglycans (PGs) consist of protein cores covalently linked to long, unbranched and highly-charged glycosaminoglycan (GAG) chains,^9^ with a multitude of functions in normal and diseased tissues.^10^ Owing to their polyanionic nature, GAGs can form interfibrillar supramolecular assemblies, capable of absorbing water into the bone extracellular matrix, thereby playing pivotal roles in maintaining osmotic pressure.^11–13^

Our previous work has shown the coupling effects of PGs and GAGs with the hydration status of bone extracellular matrix, thereby influencing the bone toughness. In vitro studies using human cadaveric bone specimens show that water functions as a plasticizer in bone only when GAGs are present,^14^ and aged-related deterioration of bone toughness is associated with a diminishing amount of GAGs and bound water in the bone matrix.^15^ Decorin (Dcn) and biglycan (Bgn), both classified as class I small leucine-rich proteoglycans (SLRP), are recognized as the predominant proteoglycan subtypes in mineralized bone tissues.^16^ They carry one or two O-linked dermatan sulfate or chondroitin sulfate GAG chains, respectively. Pioneering work by Xu et al. has demonstrated that *Bgn*-deficient mice exhibit age-dependent osteoporosis,^17^ accompanied by structural abnormalities in bone collagen fibrils.^18^ Dcn is the closest homolog to Bgn, as it shares 57% homology at the amino acid level.^19,20^ In contrast, mice with a targeted disruption of the *Dcn* gene exhibit fragile skin with markedly reduced tensile strength.^21^ Despite the alterations in collagen fibril size and shape in bone, no evident changes in bone mass were observed in *Dcn* knockout (KO) mice.^18^ The structural similarity between Bgn and Dcn suggests the existence of a compensatory mechanism, wherein the ablation of one is likely to induce an increased expression of the other.^22,23^ Consistent with this, *Bgn*/*Dcn* dKO mice exhibit more pronounced skin and bone phenotypes than those observed with the ablation of either gene alone, suggesting a synergistic function between Bgn and Dcn.^18^ However, the distinct roles of Bgn and Dcn in bone structure, water retention, fracture toughness, and in situ mechanical behaviors remain largely unclear.

Using the *Bgn*-deficient mouse model, we previously discovered the crucial role of Bgn and chondroitin sulfate in conferring toughness to bone through the retention of bound water.^24^ To investigate the specific roles between Bgn and Dcn, we generated *Bgn*/*Dcn* dKO mice in this study, overcoming the extremely low frequency of double homozygous KO mice.^18^ In addition to *Bgn* homozygous KO (*Bgn* HO) and *Dcn* homozygous (*Dcn* HO), *Dcn* heterozygous KO (*Dcn* HT) and *Bgn* HO/*Dcn* HT mice were also studied. We performed biochemical, biophysical, and biomechanical experiments to determine the GAGs, bone structure, bound water, and fracture toughness changes in bone matrix. We further used high-resolution atomic force microscopy to determine changes in the in situ mechanical behavior of extrafibrillar matrix and mineralized collagen fibril. Comparing single and dKO mice, our findings dissect the distinct roles of these two proteoglycans. Bgn plays a predominant role in maintaining bone structure, retaining bound water, and regulating fracture toughness. Furthermore, our study underscores the essential contributions of both Bgn and Dcn in enhancing the plasticity and toughness of bone, particularly in the extrafibrillar matrix region.

## Results

### *Biglycan*/*Decorin* deficiency leads to decreased body weight and reduction of total glycosaminoglycans in the bone matrix

**Fig. 1:**
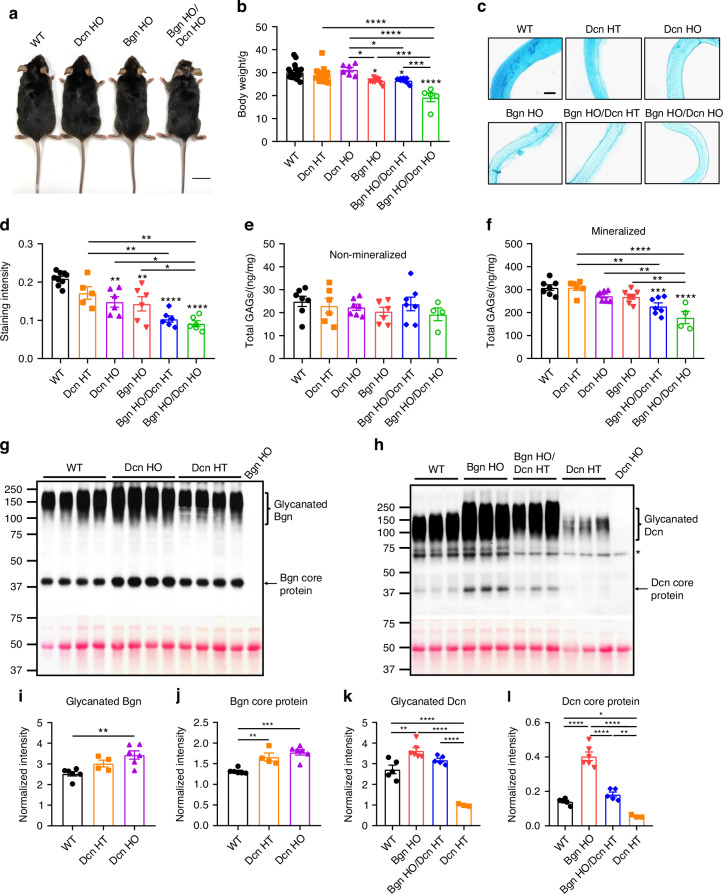
Reduction of total GAGs amount in bone of *Bgn*/*Dcn* single or double KO mice. **a** Representative image showing the body size of WT, *Dcn* KO, *Bgn* KO and *Bgn*/*Dcn* double KO mice at 6-month-old. Scale bar, 2 cm. **b** Summarized graphs of body weight of WT and *Bgn*/*Dcn* single or double KO mice at 6-month-old. WT, *n* = 18; *Dcn* HT, *n* = 16; *Dcn* HO, *n* = 6; *Bgn* HO, *n* = 9; *Bgn* HO/*Dcn* HT, *n* = 8; *Bgn* HO/*Dcn* HO, *n* = 5. **c** Representative images and (**d**) quantifications of alcian blue staining intensity of bone slices. Scale bar, 100 μm. WT, *n* = 9; *Dcn* HT, *n* = 5; *Dcn* HO, *n* = 6; *Bgn* HO, *n* = 6; *Bgn* HO/*Dcn* HT, *n* = 6; *Bgn* HO/*Dcn* HO, *n* = 6. **e**, **f** Cortical bone diaphyses were isolated with soft tissues and bone marrow removed. Total GAGs were extracted from non-mineralized and mineralized portions and determined by DMMB assay. WT, *n* = 7; *Dcn* HT, *n* = 6; *Dcn* HO, *n* = 7; *Bgn* HO, *n* = 7; *Bgn* HO/*Dcn* HT, *n* = 7; *Bgn* HO/*Dcn* HO, *n* = 4. **g** Proteoglycans from WT, *Dcn* HT, and *Dcn* HO mice mineralized bone matrix were analyzed by western blots probed with anti-Bgn antibody (upper panel). Ponceau S staining showed sample loading (lower panel). **h** Proteoglycans from WT, *Bgn* HO, *Bgn* HO/*Dcn* HT, and *Dcn* HT mice mineralized bone matrix were analyzed by western blots probed with anti-Dcn antibody (upper panel). Ponceau S staining showed sample loading (lower panel). The asterisk (*) denotes non-specific bands. **i** Glycanated Bgn and (**j**) Bgn core protein bands intensity was quantified and normalized to Ponceau S staining. WT, *n* = 6; *Dcn* HT, *n* = 4; *Dcn* HO, *n* = 6. **k** Glycanated Dcn and (**l**) Dcn core protein bands intensity was quantified and normalized to Ponceau S staining. WT, *n* = 5; *Bgn* HO, *n* = 6; *Bgn* HO/*Dcn* HT, *n* = 5; *Dcn* HT, *n* = 3. Data are presented as mean ± SEM. One-way ANOVA with Tukey test was used for statistical analysis. *, *P* < 0.05; **, *P* < 0.01; ***, *P* < 0.001; ****, *P* < 0.000 1. Asterisks above the bar graphs denote statistical comparisons with the WT groups

The body weights of WT, *Dcn* HT, *Dcn* HO, *Bgn* HO, *Bgn* HO/*Dcn* HT, and *Bgn* HO/*Dcn* HO mice were assessed. Notably, at 2 months of age, *Bgn*/*Dcn* dKO mice, including *Bgn* HO/*Dcn* HT and *Bgn* HO/*Dcn* HO groups, started to exhibit lower body weights compared to WT and single KO mice (Fig. S1A). This difference was sustained at 4 (Fig. S1B) and 6 (Fig. 1B) months of age in dKO mice, while *Bgn* HO mice also displayed a significant reduction in body weight when compared to WT and *Dcn* KO mice (Fig. 1b, S1B). As shown in Fig. 1a, *Bgn* HO/*Dcn* HO mice exhibited a notably smaller body size, with Bgn HO mice showing a slightly reduced body size compared to WT mice. However, *Dcn* deficiency did not induce body weight changes (Fig. 1a, b, S1A, B). Given that Bgn and Dcn are major subtypes of proteoglycans in the bone matrix,^16,25^ we next examined the GAGs changes through Alcian blue staining using bone sections obtained from the mid-diaphysis. The Alcian blue staining revealed the presence of GAGs throughout the bone matrix (Fig. 1c). The quantification of Alcian blue staining demonstrated a notable decrease in GAG levels in both single and dKO mice, except for *Dcn* HT mice (Fig. 1d). To further validate the alterations in GAG levels resulting from *Bgn* and *Dcn* deficiency, we determined the level of total GAGs by DMMB assay in both mineralized and non-mineralized compartments of the bone. GAGs were predominantly localized in the mineralized region of the bone matrix, exhibiting a difference of more than 10 fold compared to the non-mineralized segments (Fig. 1e, f). In comparison to WT mice, the single KO of *Dcn* or *Bgn* showed a trend of reduction in mineralized GAGs, while the dKO mice showed a significant reduction. Conversely, no notable difference was observed in the non-mineralized compartment (Fig. 1e, f).

We next analyzed the expression of Bgn and Dcn under *Dcn* and *Bgn* deficiency conditions, respectively. PGs isolated from the bone mineral matrix of 6-month-old WT, *Dcn* HT, and *Dcn* HO mice were subjected to western blotting using the anti-Bgn antibody (Fig. 1g), with Ponceau S staining showing the total protein loaded. We observed changes in GAG sizes through band shifts of glycanated PGs in the western blot. In the *Bgn* HO and *Bgn* HO/*Dcn* HT groups, the glycanated Dcn band was larger than in the WT and *Dcn* HT groups (Fig. 1h), indicating an increase in GAG size following *Bgn* KO. A similar increase in Bgn GAG size was observed in *Dcn* HO mice compared to WT mice (Fig. 1g). Notably, we observed a dose-dependent increase in both glycanated and non-glycanated forms of Bgn in *Dcn* HT and *Dcn* HO mice (Fig. 1i, j). There was a 1.36-fold increase in glycanated Bgn protein and a 1.35-fold increase in Bgn core protein when comparing *Dcn* HO and WT mice. Similarly, we determined the band intensities of glycanated and non- glycanated Dcn protein (Fig. 1h). Compared to WT mice, *Bgn* HO mice demonstrated a 1.33-fold and 2.86-fold increase in glycanated and non-glycanated forms of Dcn protein, respectively (Fig. 1k, l). Moreover, compared to *Dcn* HT mice, *Bgn* HO/*Dcn* HT mice also showed significantly elevated levels of glycanated and non-glycanated Dcn. These results suggest that the total GAGs amount in the mineralized bone matrix decreases in the absence of Bgn or Dcn, and this decrease is more pronounced in dKO mice. Furthermore, the deletion of *Bgn* or *Dcn* induces an upregulation in the expression of each other, suggesting the presence of a partial compensatory mechanism. Interestingly, the Dcn was drastically upregulated in *Bgn* KO mice, while Bgn is increased to a lesser degree in *Dcn* KO mice.

### Biglycan/Decorin deficiency leads to alterations in cortical/trabecular bone structures and bone composition

**Fig. 2:**
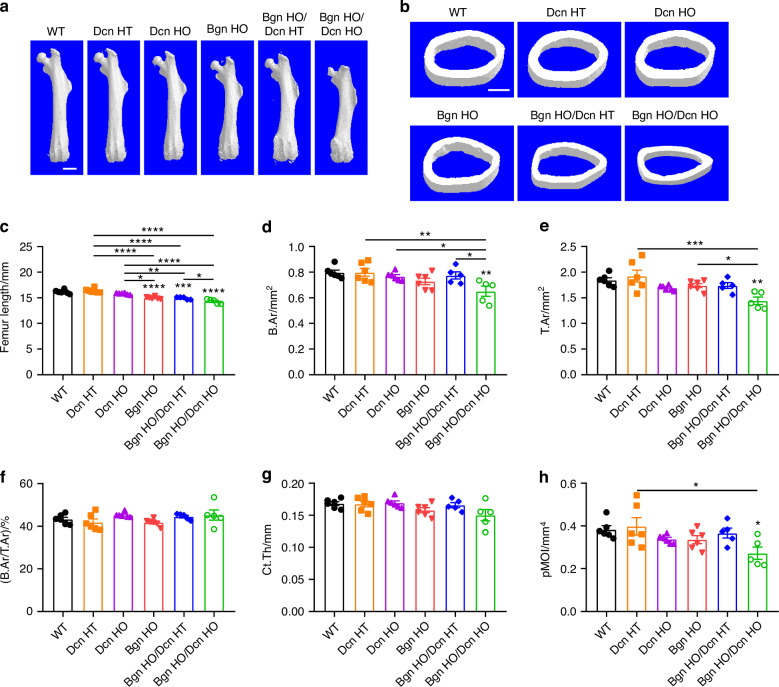
Reduced cortical bone area and polar moment of inertia, with shorter femur in *Bgn*/*Dcn* double KO mice. **a** Representative microCT images of whole femur from WT and *Bgn*/*Dcn* single or double KO mice. Scale bar, 2 mm. **b** Representative microCT images of femoral midshaft cortical bone cross-sections. Scale bar, 500 μm. **c** Summarized graphs of femur length of WT and *Bgn*/*Dcn* single or double KO mice. WT, *n* = 6; *Dcn* HT, *n* = 6; *Dcn* HO, *n* = 6; *Bgn* HO, *n* = 6; *Bgn* HO/*Dcn* HT, *n* = 5; *Bgn* HO/*Dcn* HO, *n* = 5. 3D µCT analysis of (**d**) B.Ar, (**e**) T.Ar, (**f**) B.Ar/T.Ar, (**g**) Ct.Th, and (**h**) pMOI of femoral midshaft cortical bone were shown. WT, *n* = 6; *Dcn* HT, *n* = 6; *Dcn* HO, *n* = 6; *Bgn* HO, *n* = 6; *Bgn* HO/*Dcn* HT, *n* = 5; *Bgn* HO/*Dcn* HO, *n* = 5. Data are presented as mean ± SEM. One-way ANOVA with Tukey test was used for statistical analysis. *, *P* < 0.05; **, *P* < 0.01; ***, *P* < 0.001; ****, *P* < 0.000 1. Asterisks above the bar graphs denote statistical comparisons with the WT groups. T.Ar total area, B.Ar bone area, Ct.Th cortical thickness, pMOI polar moment of inertia

We next conducted three-dimensional micro-computed tomography (μCT) analysis on the femurs of all the various genetic cohorts. We found that, *Bgn* HO, *Bgn* HO/*Dcn* HT and *Bgn* HO/*Dcn* HO mice displayed a significant reduction in femur length compared to both WT and *Dcn* KO mice (Fig. 2a–c). Notably, *Bgn* HO/*Dcn* HO mice exhibited a change in the shape. Cortical bone structure μCT analysis was conducted at the femoral midshaft (Fig. 2b). The results showed that *Bgn* HO/*Dcn* HO mice had a smaller bone area (B.Ar) and total area (T.Ar) (Fig. 2d, e), while the bone area fraction (B.Ar/T.Ar) did not show appreciable differences among the WT and KO groups (Fig. 2f). Additionally, there was a trend of reduction in *Bgn* HO and *Bgn* HO/*Dcn* HO cortical thickness (Ct.Th) compared to WT mice (Fig. 2g). The torsional rigidity, as assessed by the polar moment of inertia (pMOI), exhibited a decline in *Bgn* HO/*Dcn* HO mice compared to WT and *Dcn* HT mice (Fig. 2h).

**Fig. 3:**
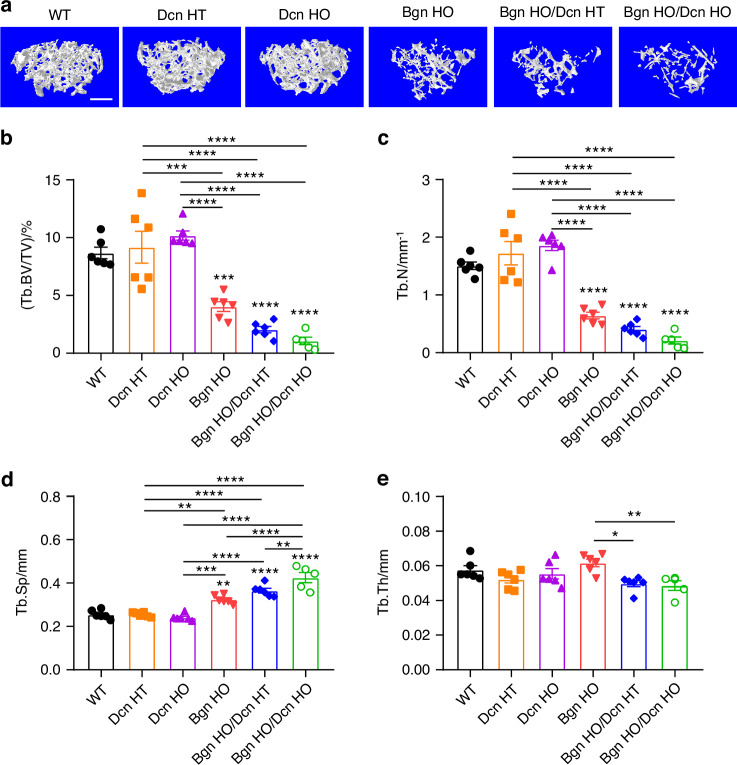
Significant decrease in femur trabecular bone mass with *Bgn* and *Bgn*/*Dcn* deficiency. **a** Representative 3D models of the metaphyseal trabecular bone for all groups. Scale bar, 500 μm. 3D microCT analyses of (**b**) BV/TV, (**c**) Tb.N, (**d**) Tb.Sp, and (**e**) Tb.Th of femoral trabecular bone were performed. Medial expansion in distal femur region of *Bgn*/*Dcn* double KO mice was excluded for analyses. WT, *n* = 6; *Dcn* HT, *n* = 6; *Dcn* HO, *n* = 6; *Bgn* HO, *n* = 6; *Bgn* HO/*Dcn* HT, *n* = 6; *Bgn* HO/*Dcn* HO, *n* = 5. Data are presented as mean ± SEM. One-way ANOVA with Tukey test was used for statistical analysis. *, *P* < 0.05; **, *P* < 0.01; ***, *P* < 0.001; ****, *P* < 0.000 1. Asterisks above the bar graphs denote statistical comparisons with the WT groups. BV bone volume, TV tissue volume, BV/TV bone volume fraction, Tb.N trabecular number, Tb.Th trabecular thickness, Tb.Sp trabecular separation

Interestingly, we observed a medial expansion structure in the distal femoral metaphysis of both *Bgn* HO/*Dcn* HT and *Bgn* HO/*Dcn* HO mice, which displays dense trabecular architecture (Fig. S2A, yellow arrows). Given the structural differences between the expanded part and the distal femur trabecular bone, μCT analysis of the trabecular bone was conducted in two distinct regions of interest (ROI). Figure 3a shows the representative μCT images of femoral metaphyseal trabecular bone, excluding the expanded regions. The analysis revealed lower trabecular bone mass in *Bgn* KO and *Bgn*/*Dcn* dKO mice compared to WT and *Dcn* KO mice (Fig. 3b–d). Moreover, there was a pronounced reduction in BV/TV and Tb.N in *Bgn*/*Dcn* dKO mice compared to *Bgn* KO mice, along with an increase in Tb.Sp (Fig. 3b–d). Additionally, the Tb.Th of *Bgn*/*Dcn* dKO mice was significantly lower than *Bgn* KO mice (Fig. 3e). The analysis of the entire trabecular bone regions within the cortical shell revealed notable decreases in bone volume fractions (BV/TV) and trabecular number (Tb.N) in *Bgn* KO and *Bgn*/*Dcn* dKO mice compared to the WT and *Dcn* KO groups, accompanied by an increase in trabecular separation (Tb.Sp) (Fig. S2B–D). Additionally, the trabecular thickness (Tb.Th) of *Bgn* HO/*Dcn* HO mice was greater compared to both *Dcn* HT and *Dcn* HO mice (Fig. S2E). In summary, the deficiency of *Bgn* and *Bgn*/*Dcn* leads to a shortened femur and a significant reduction in trabecular bone mass compared to the WT and *Dcn* KO groups. However, the alterations in cortical bone structure are relatively minimal.

In addition, bone composition was assessed by Raman spectrometry. All single and dKO groups showed significantly higher mineral/matrix ratio of v_1_PO_4_/Amide I compared to WT mice (Fig. S3A). The v_1_PO_4_/CH_2_-wag ratio, another indicator of mineral/matrix, was increased in *Dcn* HO, *Bgn* HO, and *Bgn* HO/*Dcn* HT mice (Fig. S3B). *Bgn*/*Dcn* dKO mice exhibited higher crystallinity than WT (Fig. S3C). Interestingly, the μCT analysis of tissue mineral density (TMD) revealed lower TMD in *Dcn* KO mice compared to *Bgn* KO mice, while *Bgn*/*Dcn* dKO mice had significantly higher TMD than WT mice (Fig. S3D).

### *Biglycan/Decorin* deficiency decreases the amount of bound water and fracture toughness

**Fig. 4:**
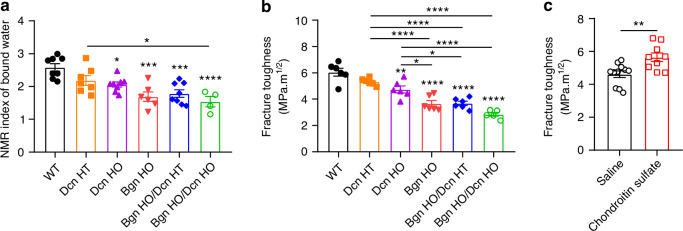
*Bgn*/*Dcn* deletion reduces bound water retention and fracture toughness in bone, while chondroitin sulfate supplementation increases fracture toughness in WT mice. **a** Low field NMR showed significant reduction of bound water amount in *Dcn* KO, *Bgn* KO and *Bgn*/*Dcn* double KO mice bone compared to WT. WT, *n* = 8; *Dcn* HT, *n* = 7; *Dcn* HO, *n* = 8; *Bgn* HO, *n* = 6; *Bgn* HO/*Dcn* HT, *n* = 8; *Bgn* HO/*Dcn* HO, *n* = 4. **b** Fracture toughness tests showed that *Bgn*/*Dcn* single or double KO led to significant reduction of bone toughness. WT, *n* = 6; *Dcn* HT, *n* = 6; *Dcn* HO, *n* = 6; *Bgn* HO, *n* = 6; *Bgn* HO/*Dcn* HT, *n* = 6; *Bgn* HO/*Dcn* HO, *n* = 5. **c** Bone fracture toughness determined in10-month-old WT mice injected with saline or chondroitin sulfate. Injections were given once a week for four weeks. Saline, *n* = 11; Chondroitin Sulfate, *n* = 9. Data are presented as mean ± SEM. *T* test and one-way ANOVA with Tukey test was used for statistical analysis. *, *P* < 0.05; **, *P* < 0.01; ***, *P* < 0.001; ****, *P* < 0.000 1. Asterisks above the bar graphs denote statistical comparisons with the WT groups

Our previous work has shown the coupling effect of proteoglycans and bound water in maintaining human bone mechanical properties during the aging process.^14,15^ To investigate the impact of *Bgn* and *Dcn* deficiency on the presence of bound water, we conducted low-energy ^1^H NMR measurements on the cortical bone of the various genetic groups. As shown in Fig. 4a, there were significant reductions in the amount of bound water in both *Bgn*/*Dcn* single KO and double KO mice compared to WT and *Dcn* HT mice. The *Bgn* HO and *Bgn* HO/*Dcn* HT mice had comparable level of bound water in the bone matrix (1.69 ± 0.14 and 1.78 ± 0.11, respectively), while there was a further decrease of bound water in the *Bgn* HO/*Dcn* HO mice (1.54 ± 0.16).

We then investigated bone fracture toughness determined by three-point flexural tests of femurs with a micro-notch in the mid-diaphysis. The fracture toughness results corresponded with bound water levels, as both *Bgn* and *Dcn* deficiencies resulted in a significant reduction in fracture toughness, with the most substantial decrease observed in *Bgn* HO/*Dcn* HO mice (Fig. 4b). Notably, there was a significant reduction in *Bgn* HO and *Bgn*/*Dcn* dKO mice compared to *Dcn* HO mice. We further determine if supplementing chondroitin sulfate would improve fracture toughness. Our previous study demonstrated that administering intradermal injections of chondroitin sulfate once a week for four weeks to WT mice significantly increased GAGs levels by 29% and bound water content by 20%.^12^ In this study, we found that the supplementation of GAGs led to significantly enhanced fracture toughness in bone (Fig. 4c). Collectively, our results indicate that a genetic background lacking Bgn and/or Dcn is linked to reduced bone matrix-bound water and deterioration in fracture toughness, and these affects can be partly reversed by GAG supplementation.

### *Biglycan*/*Decorin* KO mice exhibits altered in situ mechanical properties under the wet condition in the extrafibrillar matrix

**Fig. 5:**
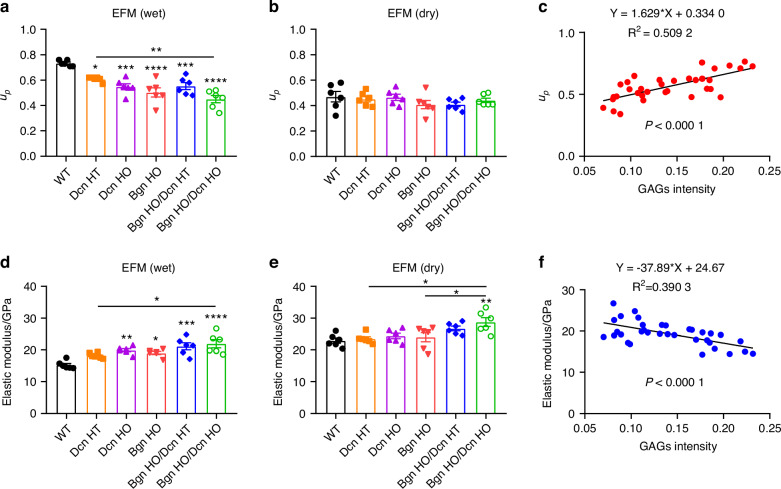
Altered in situ mechanical properties of *Bgn*/*Dcn* single or double deficiency under hydrated cobndition by nanoindentation measurements using high-resolution AFM indicated. **a**, **b** In the compartment of extrafibrillar matrix (EFM), permanent energy dissipation (*u*_*p*_) was determined in WT, *Dcn* KO, *Bgn* KO and *Bgn*/*Dcn* double KO mice when water is present or under the dry bone condition. **c** Correlation analysis indicated that plastic deformation of bone increased significantly with the amount of GAGs in the mineralized compartment. **d**, **e** Elastic modulus was determined in WT, *Dcn* KO, *Bgn* KO and *Bgn*/*Dcn* double KO mice when water is present or under dry bone condition. WT, *n* = 6; *Dcn* HT, *n* = 6; *Dcn* HO, *n* = 6; *Bgn* HO, *n* = 6; *Bgn* HO/*Dcn* HT, *n* = 6; *Bgn* HO/*Dcn* HO, *n* = 6. **f** Correlation analysis indicated that elastic modulus of bone decreased significantly with the amount of GAGs in the mineralized compartment. One-way ANOVA with Tukey test was used for statistical analysis. Data are presented as mean ± SEM. *, *P* < 0.05; **, *P* < 0.01; ***, *P* < 0.001; ****, *P* < 0.000 1. Asterisks above the bar graphs denote statistical comparisons with the WT groups

Next, we conducted nanoindentation measurements using high-resolution atomic force microscopy (AFM) to evaluate the in situ mechanical properties of *Bgn*/*Dcn* KO mice. Mouse bone samples were examined under both wet and dry conditions. In the wet condition, bone samples were soaked in water, whereas in the dry condition, bone samples were dehydrated by heating to remove bound water from the matrix. The permanent energy dissipation capacity (*u*_*p*_) of the extrafibrillar matrix (EFM) decreased by 35.6% in WT mice when shifting from a hydrated to a dry bone condition (Fig. 5a, b). When comparing the *Bgn*/*Dcn* KO and WT groups, there was a significant reduction in tissue-level permanent energy dissipation capacity across all KO groups, with the most substantial decrease in dKO groups (Fig. 5a). However, these differences were abolished following dehydration (Fig. 5b). Notably, the *Bgn* HO/*Dcn* HO group exhibited comparable permanent energy dissipation capacity values in both wet and dry conditions, measuring 0.45 ± 0.03 and 0.44 ± 0.02, respectively. These findings suggest that *Bgn* HO/*Dcn* HO mice may have completely lost their capacity for water retention. Additionally, a strong positive linear correlation (*P* < 0.000 1) was observed between the GAGs intensity determined by Alcian blue staining and in situ permanent energy dissipation capacity (Fig. 5c). Furthermore, we assessed the elastic modulus using AFM in both wet and dry conditions for the WT and *Bgn*/*Dcn* single and double KO groups. As shown in Fig. 5d, e, the removal of bound water resulted in an elevation of elastic modulus in WT mice (15.25 ± 0.51 under wet condition vs. 22.90 ± 0.81 under dry condition). There was an increase of elastic modulus in *Bgn*/*Dcn* single and double KO mice compared to WT mice under the hydrated condition (Fig. 5d), while the differences were not evident after bound water removal, except in the case of *Bgn* HO/*Dcn* HO mice, which maintained the elevated levels (Fig. 5E). Moreover, we identified a significant negative linear correlation (*P* < 0.000 1) between the GAGs amount and in situ elastic modulus of EFM (Fig. 5f). These findings suggest that *Bgn*/*Dcn* deficiency and water had a coupling effect on the in situ permanent energy dissipation and elastic modulus of the EFM within bone.

### *Biglycan*/*Decorin* KO mice exhibits disorganization in mineralized collagen fibrils with minimal in situ mechanical property changes

**Fig. 6:**
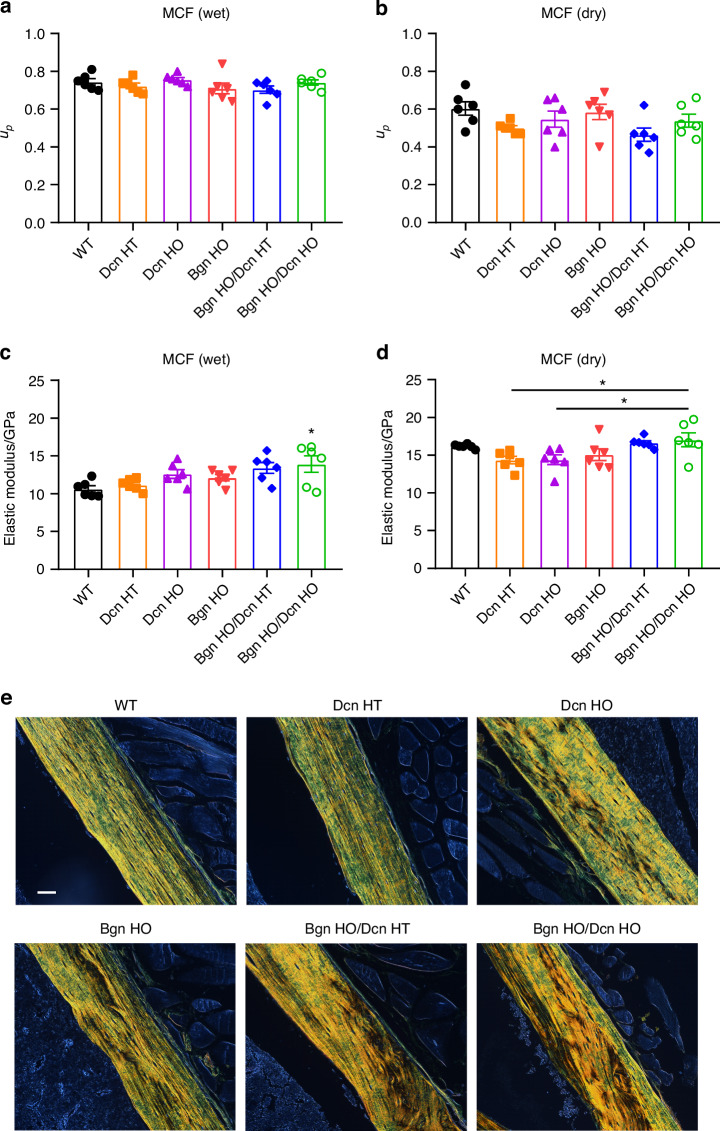
Increased elastic modulus in mineralized collagen fibrils and altered collagen structure in *Bgn*/*Dcn* double KO mice. **a**, **b** In the compartment of mineralized collagen fibrils (MCF), permanent energy dissipation (*u*_*p*_) was determined in WT, *Dcn* KO, *Bgn* KO and *Bgn*/*Dcn* double KO mice when water is present or under dry bone condition. **c**, **d** Elastic modulus was determined in WT, *Dcn* KO, *Bgn* KO and *Bgn*/*Dcn* double KO mice when water is present or under dry bone condition. WT, *n* = 6; *Dcn* HT, *n* = 6; *Dcn* HO, *n* = 6; *Bgn* HO, *n* = 6; *Bgn* HO/*Dcn* HT, *n* = 6; *Bgn* HO/*Dcn* HO, *n* = 6. Data are presented as mean ± SEM. One-way ANOVA with Tukey test was used for statistical analysis. *, *P* < 0.05; **, *P* < 0.01; ***, *P* < 0.001; ****, *P* < 0.000 1. Asterisks above the bar graphs denote statistical comparisons with the WT groups. **e** Femoral midshaft cortical bone sections from WT, *Dcn* KO, *Bgn* KO and *Bgn*/*Dcn* double KO mice were prepared. Collagen organization was assessed using picrosirius red staining under polarized light. Scale bar, 50 μm

Using AFM measurements, we determined the permanent energy dissipation capacity (*u*_*p*_) and elastic modulus from the mineralized collagen fibril (MCF) region. In contrast to the EFM, the permanent energy dissipation capacity of MCF did not show any appreciable change among the various KO groups and WT mice under wet condition (Fig. 6a). After bound water removal, there was a general decrease in permanent energy dissipation capacity. However, no significant differences were noted among the different KO groups (Fig. 6b). The results for elastic modulus demonstrated that, under wet conditions, *Bgn* HO/*Dcn* HO mice exhibited significantly higher levels than WT mice (Fig. 6c). Dehydration led to a general increase in the elastic modulus in MCF, with a significant increase in *Bgn* HO/*Dcn* HO mice compared to *Dcn* KO mice (Fig. 6d).

To determine if these differences were associated with structural changes in collagen fibrils, we investigated the organization of collagen fibrils using picrosirius red staining, which enhances the natural birefringence of collagen when subjected to polarized light. As depicted in Fig. 6e, the picrosirius red staining patterns for WT and *Dcn* HT mice appear similar. In contrast, collagen network disorganization is evident in *Bgn*/*Dcn* single and dKO mice, with more pronounced disorganization observed in the dKO groups. Furthermore, there is a prevalence of yellow-red birefringence in the *Bgn* HO, *Bgn* HO/*Dcn* HT, and *Bgn* HO/*Dcn* HO groups in comparison to WT mice, indicating the presence of fewer immature collagen fibers (Fig. 6e). Taken together, these findings reveal noticeable disorganization in the structure of mineralized collagen fibrils in *Bgn*/*Dcn* KO mice, while the changes of the in situ permanent energy dissipation and elastic modulus of the MCF are minimal.

### Activation of ERK and p38 MAPK signaling pathways was markedly impaired in *Biglycan*/*Decorin* KO mice

**Fig. 7:**
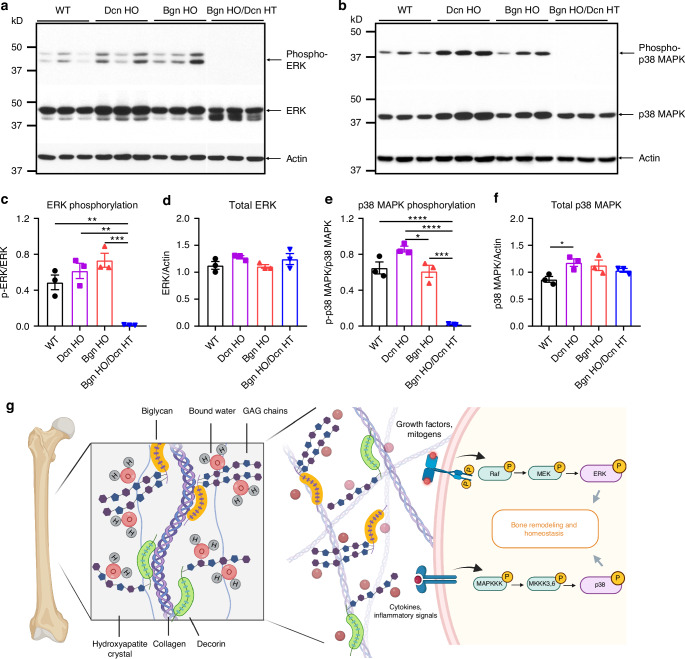
Phosphorylation of ERK and p38 MAPK signaling pathways was markedly impaired in *Bgn*/*Dcn* double KO mice. Whole bone lysates extracted from the cortical bones of WT, *Dcn* HO, *Bgn* HO, and *Bgn* HO/*Dcn* HT mice were analyzed by western blot using antibodies against (**a**) phosphorylated ERK (p-ERK), total ERK, and β-actin, and (**b**) phosphorylated p38 MAPK (p-p38 MAPK), total MAPK, and β-actin. **c** ERK phosphorylation was determined by normalizing the intensity of p-ERK protein bands to total ERK. **d** Total ERK was determined by normalizing the intensity of ERK protein bands to β-actin. **e** p38 MAPK phosphorylation was quantified by normalizing the intensity of p-p38 MAPK protein bands to total p38 MAPK. *n* = 3 mice/group. **f** Total p38 MAPK was determined by normalizing the intensity of p38 MAPK protein bands to β-actin. **g** Schematic summary of the roles of Bgn and Dcn in water retention and the activation of ERK and p38 MAPK signaling pathways (modified from^13,24^). Dcn and Bgn are characterized by one or two chondroitin sulfate/dermatan sulfate chains attached to horseshoe-shaped core proteins, respectively. The GAG chains are highly negatively charged, which play a pivotal role in retaining bound water, thus conferring ductility and plasticity to bone. Bgn and Dcn have been reported to interact with various cytokines, growth factors, and cell surface receptors to form stable complexes, thereby activating downstream pathways that regulate bone homeostasis.^13,26,27^ Specifically, our data suggest that Bgn and Dcn are indispensable for the activation of ERK and p38 MAPK signaling pathways, which may explain the compensatory response observed between these two PGs. Figure created using BioRender (https://biorender.com/). Data are presented as mean ± SEM. One-way ANOVA with Tukey test was used for statistical analysis. *, *P* < 0.05; **, *P* < 0.01; ***, *P* < 0.001; ****, *P* < 0.000 1

To investigate the underlying biological processes involved in the compensatory mechanism between Bgn and Dcn, we performed western blot analysis using bone lysates from *Bgn*/*Dcn* single and double KO mice. As shown in Fig. 7a–f, Bgn and Dcn are essential for the phosphorylation and activation of ERK and p38 MAPK signaling pathways. Specifically, *Bgn* HO and *Dcn* HO mice exhibited a slight trend toward higher ERK phosphorylation levels, which was completely abolished in *Bgn* HO/*Dcn* HT mice (Fig. 7c). Notably, total ERK protein levels remained unchanged across WT, *Bgn*/*Dcn* single and double KO mice (Fig. 7d). Similarly, p38 MAPK phosphorylation was significantly inhibited in *Bgn* HO/*Dcn* HT mice (Fig. 7e), while both phosphorylated and total p38 MAPK levels were elevated in *Dcn* HO mice (Fig. 7e, f). This suggests that Bgn may play a more critical role in regulating p38 MAPK levels and activation, particularly since Bgn was upregulated in *Dcn* HO mice.

## Discussion

In the current study, we have identified Bgn and Dcn as two proteoglycans playing distinct and indispensable roles in regulating GAG content, bone microstructure, bone mass, bound water amount, mechanical properties, and ERK and p38 MAPK signaling pathways. Ablation of *Bgn* or *Dcn* gene induces overexpression of the other in bone tissue, suggesting the presence of a partial compensatory mechanism. Trabecular bone loss was observed in *Bgn* but not *Dcn* KO mice. Additionally, *Bgn* deficiency resulted in a more profound decrease in bound water and fracture toughness than Dcn KO mice, suggesting Bgn’s predominant role in maintaining bone structure, water retention, and fracture toughness. Compared to *Bgn* or *Dcn* single KO groups, more profound differences were observed in *Bgn*/*Dcn* dKO mice. Moreover, deficiency of *Bgn* and/or *Dcn* significantly reduced the permanent energy dissipation capacity (*u*_*p*_) of EFM when water is present, while having limited effects on MCF, highlighting the coupling of Bgn/Dcn-attached GAGs and water in toughening the EFM of bone. We also found that Bgn and Dcn are indispensable for the activation of ERK and p38 MAPK signaling pathways, which may explain the compensatory response observed between these two PGs. As summarized in Fig. 7g, Dcn and Bgn are characterized by one or two chondroitin sulfate/dermatan sulfate chains attached to horseshoe-shaped core proteins, respectively. These highly negative charged GAG chains play a pivotal role in retaining bound water, thereby conferring ductility and plasticity to bone. Bgn and Dcn have been reported to interact with various cytokines, growth factors, and cell surface receptors to form stable complexes, thereby activating downstream pathways that regulate bone homeostasis.^13,26,27^ Consistent with our findings, previous studies have shown that Bgn mediates ERK phosphorylation and signal transduction through the transcription factor Runx2,^28^ while Dcn acts as a ligand for the epidermal growth factor receptor (EGFR), which is responsible for ERK activation.^29^ Furthermore, both Bgn and Dcn have been implicated in activating p38 MAPK signaling through Toll-like receptors.^30,31^

Bgn and Dcn both belong to the class I SLRP family, which is the most abundant type of PGs in the bone matrix. SLRPs are characterized by 36-42 kD protein cores with 10-12 leucine-rich repeats. With the horseshoe-shaped protein core structure, they interact with a multitude of growth factors and cytokines,^32^ and regulate cell proliferation, matrix deposition, and tissue remodeling.^27,33–35^ Our results suggest that mice lacking Bgn display a smaller body size and lower body weight, which are not observed in *Dcn* KO mice. Using μCT analysis, our findings reveal that *Bgn*-deficient mice have a shorter femur, with significantly reduced femoral trabecular bone mass. In contrast, *Dcn*-deficient mice show no changes in bone microstructure. These phenotype differences in *Bgn* KO and *Dcn* KO mice could be attributed to several aspects. First, the distinct spatial expression patterns of Dcn and Bgn in mature and immature bones suggest varied roles in osteoid formation and mineralization. In immature bones, Dcn is expressed throughout the osteoid matrix and associated with periosteal layers, while in mature bone, it is found in the perilacunar matrix and osteocyte canaliculi. Conversely, Bgn, initially localizing to osteocyte lacunae walls, is evenly distributed throughout the mature bone matrix, potentially acting as a shear force transducer.^16,23,36^ In addition, these two SLRPs have different tissue localization and abundance, sometimes even being mutually exclusive.^16^ It has been reported that Dcn is the most highly expressed PGs in the skin,^37^ which explains why Dcn KO mice exhibit more prominent skin phenotypes.^21^ Secondly, Bgn and Dcn exhibit different binding affinities to mineral and soluble matrix constituents. For example, the affinity of Bgn for hydroxyapatite is greater than that of Dcn, suggesting that Bgn plays a more prominent role in regulating mineralization.^38^ While both *Bgn* and *Dcn* deficiency impact bone collagen fibril size and shape, the effects are opposite. *Bgn* KO mice show an increase in fibril diameter, whereas the fibrils size range decrease in *Dcn* KO mice.^18^ Our results also indicate opposing regulation of TMD in *Dcn* KO and *Bgn* KO mice.

In this study, male mice were utilized due to the minimal effects of Bgn deficiency on bone metabolism observed in female mice. In a previous study by Nielsen et al.,^39^ male and female Bgn KO mice were studied and compared for bone phenotypes. Interestingly, in contrast to the male Bgn KO mice which showed age-related osteoporosis, female Bgn KO mice exhibited only minimal effects on basal bone turnover and bone mass, indicating a gender difference. Similar sexually dimorphic responses in bone have been reported in other mouse models as well.^40–42^ One possible reason for this gender difference is that Bgn gene is located on the X chromosome, and its knockout may differently influence gene expression related to bone growth and metabolism in males and females. The detailed mechanism still warrants further investigation.

We found that the phenotypes exhibited by *Bgn* KO mice are more profound than that detected in *Dcn* KO mice. One possible explanation is due to the compensatory effects. By examining the protein expression of Bgn and Dcn in *Dcn* and *Bgn* KO mice, respectively, we found that unexpectedly, Dcn was drastically upregulated in *Bgn* KO, while Bgn is increased to a lesser degree in *Dcn* KO. Thus, we conclude that the bone phenotype observed in *Bgn* KO mice cannot be compensated by Dcn proteoglycan overexpression, suggesting that biglycan plays a uniquely positive role in bone structure, water retention, and fracture toughness. Compensatory upregulation of *Bgn* gene expression has been observed in the cornea of *Dcn*-deficient mice.^43^ However, this compensatory response was not observed in the articular cartilage,^44^ indicating that the compensatory mechanisms between these two SLRPs are tissue specific. We should point out, however, that the less obvious bone phenotypes in *Dcn* KO could be partially attributed to the upregulation of Bgn. These data further indicate that between these two proteoglycans, Bgn plays more predominant roles in bone mineral matrix. Recent studies have shown that Bgn plays a more prominent role in regulating subchondral bone structure, with its loss resulting in more significant effects on subchondral bone plate and trabecular bone structure. Conversely, Dcn loss has been shown to have more limited effects on bone but more substantial impact on cartilage degeneration during osteoarthritis progression, where it mediates physical linkages for aggrecan molecules.^44–46^ These findings align with our observations and underscore the importance of Bgn in bone homeostasis and remodeling.

To further access the essential roles of these proteoglycans, we generated *Bgn*/*Dcn* dKO mice. Due to the extremely low breeding frequency in generating *Bgn*/*Dcn* dKO mice, as described previously,^18^ to our knowledge, detailed bone analysis of these mice has not been conducted before. This dKO model becomes essential given that the single KO of one type of proteoglycan exhibited a partial compensatory expression of the other and vice versa. We provide convincing evidence that *Bgn*/*Dcn* dKO mice exhibit much more severe bone structure and mechanical property deficits than single KO mice, indicating synergetic rather than additive functions between Bgn and Dcn. Remarkably, our μCT analysis of the distal femur microstructure in *Bgn*/*Dcn* dKO mice revealed a significant medial expansion, manifesting as an osteophyte. In this study, we used conventional KO models, which showed altered bone development and growth. Robinson et al.^47^ have developed inducible Bgn and Dcn KO models using floxed lines, which could address the challenge of breeding double KO mice and provide valuable insights into the specific roles of Bgn and Dcn during aging and disease pathophysiology, while allowing for normal expression during development. Future studies incorporating these inducible models could help to elucidate the specific contributions of these proteoglycans to mature bone physiology.

Our study provides direct evidence for the mechanical contribution of PGs/GAGs in bone tissue, revealing a significant reduction in fracture toughness with *Bgn*/*Dcn* single or double deficiency, accompanied by decreased bound water. In our previous study, we identified chondroitin sulfate as the predominant subtype of GAGs in the bone matrix. Supplementing chondroitin sulfate resulted in elevated levels of GAGs and bound water in WT mice.^15,24^ In this study, we demonstrate that mice injected with chondroitin sulfate exhibit an increase in fracture toughness. Furthermore, the coupling effects of PGs/GAGs and bound water are investigated through nanoindentation measurements using high-resolution AFM. Our findings reveal that dehydration has profound effects on the in situ permanent energy dissipation capacity and elastic modulus. It has been suggested that bound water facilitates load transfer between collagen and mineral, dissipates energy, and imparts ductility to bone.^12,48^ GAGs contribute to this by conferring a negative charge. Chondroitin sulfate aggregates and functions as a ‘semi-permeable’ membrane that attracts water, sustaining the osmotic pressure in the organic compartment of bone.^49–51^ Consequently, ablation of *Bgn* and/or *Dcn* leads to significant reduction of bound water level. While there are no overt changes in bone structure of *Dcn* KO mice, fracture toughness tests and AFM measurements indicate impaired bulk and in situ mechanical competence in *Dcn* KO mice. These findings underscore that the bone quality is influenced by a combination of factors, whereas water is one of the key determinants. In addition, *Dcn* deficiency also results in altered mineral/matrix composition assessed by Raman spectrometry and decreased mineral density. These may also contribute to impaired mechanical properties after *Dcn* gene ablation. We observed a more pronounced reduction in bound water in *Bgn* KO mice compared to *Dcn* KO mice. This difference could be attributed to variations in GAG chains and their water-binding capacity. The N-terminal region of Bgn has two Ser-Gly sites for chondroitin sulfate/dermatan sulfate chain attachment, whereas Dcn carries a single chondroitin sulfate/dermatan sulfate GAG chain.^47^ Interestingly, the bound water levels in *Bgn* KO and *Bgn*/*Dcn* dKO appear comparable in NMR measurements, possibly due to changes in solid-like protons. Normalization to solid-like protons amount for the bound water index calculation is aimed to eliminate the bone size effects. However, structural solid-like protons, integral to the mineral lattice or tropocollagen ultrastructure,^52^ might have decreased along with bound water in *Bgn* HO/*Dcn* HO mice. This could lead to an underestimated reduction in the bound water index, given the substantial alterations in mineral and collagen in these mice.

Using the high-resolution AFM technique, our study is capable of measuring the in situ mechanical behavior in the compartments of MCF and EFM at nanometer scales.^53,54^ The findings suggest that loss of GAGs in KO mice specifically influence the permanent energy dissipation capacity (*u*_*p*_) of EFM, whereas such GAGs loss seem to have minimal effects on *u*_*p*_ of MCFs. The elastic modulus of the EFM exhibits a notable increase in KO mouse models, aligning with earlier observations in human bone specimens following the removal of GAGs.^54^ The in situ mechanical behavior of EFM was affected by the ablation of *Bgn* and/or *Dcn* genes, and the differences were abolished after removal of bound water. Our previous work using *Bgn* KO mice exhibited a further reduction of tissue-level toughness under dehydration condition, possibly due to the overexpression of Dcn. In the current study, *Bgn*/*Dcn* dKO mice showed comparable levels of permanent energy dissipation between wet and dry conditions, indicating the importance of Bgn and Dcn in retaining water and facilitating in situ permanent energy dissipation. Our results obtained from AFM measurements at the ultrastructure level overall align well with the outcomes of the whole femur fracture toughness test, except for the differences between *Dcn* KO and *Bgn* KO mice. A plausible explanation is that whole femur testing assesses bulk bone tissue properties, while AFM nano-indentation, with its higher nanoscale resolution, captures local variations. These techniques complement each other, providing a comprehensive understanding of bone properties at different scales. The AFM data suggest that both Bgn and Dcn are key players in the regulation of bound water and permanent energy dissipation in the EFM.

In summary, this study unveils the indispensable roles of GAGs and PGs in regulating bone microstructure, bound water content, and mechanical behavior at bulk and ultrastructural levels. Bgn deficiency induced the overexpression of Dcn, and vice versa. Ablation of both *Bgn* and *Dcn*, compared to *Bgn*/*Dcn* single KO mice, leads to more profound reduction in trabecular bone mass, GAGs amount, whole femur fracture toughness, and in situ elastic modulus, indicating a complementing function between Bgn and Dcn in bone matrix, with Bgn plays a more dominant role. Furthermore, coupling with bound water, GAGs specifically influence the permanent energy dissipation capacity (*u*_*p*_) and elastic modulus in EFM of bone. These findings highlight the pivotal role of Bgn and Dcn in regulating bone mechanical properties through retaining bound water in the bone matrix.

## Materials and methods

### Animals

The *Bgn* KO mice were generated as previously described^17^ and were generously provided by Marian F. Young (National Institutes of Health, Bethesda, MD, USA). The *Dcn* KO mice were generated as previous described^21^ and were generously provided by Renato V. Iozzo (Thomas Jefferson University). All experiments were performed using age-matched WT (*Bgn*^+/0^ *Dcn*^+/+^, *Bgn* is on X chromosome), *Bgn* HO (*Bgn*^−/0^), *Dcn* HT (*Dcn*^+/–^), *Dcn* HO (*Dcn*^–/–^), *Bgn* HO/*Dcn* HT (*Bgn*^−/0^ *Dcn*^+/–^), and *Bgn* HO/*Dcn* HO (*Bgn*^−/0^ *Dcn*^-/-^) male mice, as *Bgn* deficiency showed a minor effect on bone metabolism in female mice.^39^ Homozygous *Dcn*-deficient male mice were crossed with homozygous *Bgn*-deficient female mice. F1 female mice with the genotype *Bgn*^+/–^ *Dcn*^+/–^ were bred to heterozygous *Dcn*-deficient male mice to generate F2 animals for experiments. The genotypes were determined by a PCR-based assay.^18^ For chondroitin sulfate (CS) supplementation experiments, we chose to use middle-aged mice approximately 10 to 11 months old, based on their life phase equivalencies between humans. The dosage of 4 mg/kg CS and the intradermal route of administration were selected based on a prior study that showed incorporation of intradermally injected CS into mouse tissues.^55^ WT male mice were injected at the base of the tail with CS (Y0000280, Sigma-Aldrich, St. Louis, MO, USA) dissolved in saline and filtered. Control mice were injected with the same volume saline. Injections were given once a week for four weeks. All the mice utilized in this study were of C57BL/6 background and were housed in a temperature-controlled room with a 12-h light/dark cycle at the University of Texas Health Science Center at San Antonio (UTHSCSA) Institutional Lab Animal Research (LAR) facility. The mice had ad libitum access to food and water. All animal procedures were conducted in compliance with the National Institutes of Health guidelines for the care and utilization of laboratory animals. The animal protocols were approved by the UTHSCSA Institutional Animal Care and Use Committee.

### Extraction and quantification of glycosaminoglycans from bone tissues

The mouse femurs and tibias were isolated free of soft tissues, and bone marrow was removed by flushing with cold PBS. GAGs were extracted from both non-mineralized (e.g., osteoids, membranes, and bone surfaces) and mineralized compartments within the bone matrix.^56–58^ Briefly, cortical bone tissues were pulverized into fine powders using a mortar and pestle immersed in liquid nitrogen. The bone powders were first treated in lysis buffer I (4 mol/L guanidine HCl, 0.05 mol/L Tris, and 0.1 mol/L 6-aminocaproic acid, pH 7.4) with proteinase inhibitors at 4°C on an orbital shaker for 72 h to remove GAGs from the non-mineralized compartment of bone, and lysis buffer II (4 mol/L guanidine HCl, 0.5 mol/L EDTA tetrasodium salt, 0.05 mol/L Tris, and 0.1 mol/L 6-aminocaproic acid, pH 7.4) with proteinase inhibitors at 4 °C on an orbital shaker for 72 h to remove GAGs from the mineralized compartment of bone. The supernatant from each compartment was collected and GAGs quantification was performed using the dimethylmethylene blue (DMMB) assay, as described previously.^24^

### Western blotting

Protein concentrations of whole bone lysate or proteoglycans extracted from the bone mineralized matrix were determined by Micro BCA Protein Kit (Thermo Scientific, Rockford, IL, USA). Protein samples were boiled in SDS buffer, separated by 10% SDS-PAGE, and electroblotted onto a nitrocellulose membrane. Membranes were incubated with the following primary antibodies: Bgn (LF-159) antibody (1:500, ENH020-FP, Kerafast, Boston, USA), Dcn (LF-114) antibody (1:500, ENH019-FP, Kerafast, Boston, USA), p44/42 MAPK (Erk1/2) antibody (1:1 000, #9102, Cell Signaling Technology, Danvers, MA), phospho-p44/42 MAPK (Erk1/2) (Thr202/Tyr204) Antibody (1:1 000, #9101, Cell Signaling Technology, Danvers, MA), p38 MAPK antibody (1:1 000, #8690, Cell Signaling Technology, Danvers, MA), phospho- p38 MAPK antibody (1:1 000, #4511, Cell Signaling Technology, Danvers, MA), and β-actin antibody (1:5 000, MA515739, Thermo Scientific, Rockford, IL, USA). Primary antibodies were detected with HRP-conjugated anti-goat secondary antibody and were developed using an ECL kit (Amersham Biosciences, Piscataway, NJ, USA).

### Alcian blue staining

Alcian blue staining is a semiquantitative technique used to assess GAG contents.^59,60^ Bone slices from the mid-diaphyseal femurs of each mouse were prepared by using a precision wafering saw (PICO 155, PACE Technologies, Tucson, AZ, USA) to create 80 μm thick cross-sectional surfaces, which were then polished with P1200 grit-sized sandpaper of the PHOENIX 4000 system (Buehler, Lake Bluff, IL, USA). The bone slices were incubated in 3% acetic acid for 3 min, then stained in 1% Alcian blue 8GX (Sigma-Aldrich, St. Louis, MO, USA) for 4 h. Post-staining, the slices were destained, washed with 1×PBS three times for 5 min each, dehydrated in gradient alcohol, and cleared in Xylene. Image capture was performed using a Keyence microscope (BZ-X710, Keyence, Osaka, Japan), and the staining intensity were analyzed using NIH Image J software.

### Micro-computed tomography (μCT) measurement

The SkyScan 1172 scanner (Brüker, Kontich, Belgium) was operated under the following parameters: 59 kV voltage, 167 μA beam intensity, 0.5 mm aluminum filter, 750 ms exposure time, 0.4-degree rotation step, 4-frame averaging, 1 024 × 1 024 pixel matrix, and a 9.98 μm isotropic voxel dimension. The acquired images underwent background noise removal by eliminating disconnected objects smaller than 4 pixels in size. Three volumes of interest (VOI) were selected for analysis, two in the metaphysis and the other in the midshaft regions of the femur. In the distal femoral metaphysis, the trabecular bone VOI was positioned 50 slices proximal to the growth plate and extended 150 slices in the proximal direction. Irregular contours were manually drawn along the perimeter of the cortical bone a few pixels away from the endosteal boundary, including or excluding the medial expansion. These contours were created every 10 slices and interpolated to obtain a 3D VOI. A grayscale value threshold of 90 was set for a set of 8-bit slices. The analysis of cortical bone structure was performed over 50 slices centered at the 55% position (from proximal to distal) in the femur diaphysis. The cortex was selected using automated, density-driven contouring with a threshold of 120, for a set of 8-bit slices. The structural morphometric properties of the cortical and trabecular regions were analyzed using CT Analyser software (CTAn 1.18.8.0, Brüker, Kontich, Belgium).

### Low-energy NMR measurement of bound water in bone matrix

The bound water was measured using a low-field NMR technique. Based on the T_2_ relaxation information, the relative amount of freely mobile water, bound water, and lattice water was assessed. Briefly, a low-field NMR spectrometer (Bruker 20 MHz) was configured at a proton frequency of 20 MHz to assess the amount of bound water using bone powders from the animal models. 1H spin–spin (T_2_) relaxation profiles were obtained using the NMR CPMG,{90° [−τ − 180° − τ (echo)]n – TR} spin echo method, which has a 9.5 μs wide RF-90° pulse, τ of 1 000 μs, and TR (sequence repetition rate) of 15 s. One thousand echoes were recorded (one scan with *n* = 1 000) to obtain the T_2_ profile, with 64 scans used for the measurements. The FID signal was sampled and recorded at 2 μs intervals using a 9.5 μs wide 90° RF-pulse for bound water measurements. For each FID profile, 1 500 data points were acquired in one scan (an approximate 3 ms delay window). An inversion relaxation technique was used to invert both CPMG and FID data to a T_2_ relaxation distribution spectrum. The relative amount of bound water was then estimated as the ratio of the total intensity of bound water signal with respect to the total intensity of the solid form water signal (representative of bone mass) of each sample.

### Fracture toughness test

In the notched toughness test, both ends of the femurs were removed using a low-speed saw (PICO 155, PACE Technologies, Tucson, AZ, USA). Notches were carefully introduced through the anterior surface of the mid-diaphysis using a razor blade, followed by polishing using 1 µm MicroPolish II Suspension (40-6361-006, BUEHLER, IL, USA). The notched bones underwent a three-point bending test until catastrophic failure was reached, with a span distance of 6 mm and a constant loading rate of 0.033 mm/min. Load and deflection data were recorded for each test, with the maximum force obtained during the testing process. Following the fracture, the fracture surfaces were examined under a microscope and subsequently analyzed using Image J to quantify bone geometry, half-notch initiation angle, and cortical bone thickness. The fracture toughness (K_f_) of bone was calculated using the equation as described previously,^61^

$${\rm{K}}{\rm{f}}={\rm{F}}{\rm{b}}\frac{{PS}{R}_{0}}{\pi \left({{\rm{R}}}_{0}^{4}-{{\rm{R}}}_{{\rm{i}}}^{4}\right)}\sqrt{\pi {R}_{m}\theta }$$

where, F_b_ represents a geometric constant for thick-walled cylinders which is determined by the mean thickness and radius of the cortical bone, P is the maximum force recorded during the test, S is the span width, Rm, Ri, and Ro are the mean, inner, and outer radius of the bone, respectively, and θ is the half-crack initiation angle. The geometric constant F_b_ for thick-walled cylinders is defined as:

$${F}_{b}=\left(1+\frac{t}{2{R}_{m}}\right)\left[{A}_{b}+{B}_{b}\left(\frac{\theta }{\pi }\right)+{C}_{b}{\left(\frac{\theta }{\pi }\right)}^{2}+{D}_{b}{\left(\frac{\theta }{\pi }\right)}^{3}+{E}_{b}{\left(\frac{\theta }{\pi }\right)}^{4}\right]$$

Where

$${A}_{b}=0.651\,33-0.577\,4\varepsilon -0.342\,7{\varepsilon }^{2}-0.068\,1{\varepsilon }^{3}$$

$${B}_{b}=1.879+4.795\varepsilon +2.343{\varepsilon }^{2}-0.6197{\varepsilon }^{3}$$

$${C}_{b}=-9.779-38.14\varepsilon -6.611{\varepsilon }^{2}+3.972{\varepsilon }^{3}$$

$${D}_{b}=34.56+129.9\varepsilon +50.55{\varepsilon }^{2}+3.374{\varepsilon }^{3}$$

$${E}_{b}=-30.82-147.6\varepsilon -78.38{\varepsilon }^{2}-15.54{\varepsilon }^{3}$$

$$\varepsilon =\log \left(\frac{t}{{R}_{m}}\right)$$

with *t* being the mean thickness of the cylinder. This solution is valid for both thin-walled and thick-walled bones, specifically for 1.5<R_m_/*t* < 80.5 and for a range of half-crack angles, 0<θ/π < 0.611.

### Atomic force microscopy

The mouse femur was freshly embedded in polymethyl methacrylate (PMMA) resin. Bone slices were cut from the cross-section of the mid-diaphysis using a precision low-speed diamond saw (Pico155 precision cutter, Pace Technologies). These slices underwent a sequential lapping process with various grits of sandpapers and were further polished with 0.05 μm MicroPolish II Suspension (40-10083, Buehler, IL, USA) until achieving a thickness of approximately 400 μm and a surface roughness of less than 30 nm (Fig. S4A). The bone slice was mounted onto the hollow cylindrical holder in a custom-designed specimen chamber. For AFM measurements under wet condition, the chamber was filled with water to ensure hydration of the bone slice from underneath, facilitated by water absorption through the gauze placed inside the hollow specimen holder. For AFM measurements under the dry condition, bone slices were dehydrated in a vacuum at 60 °C for 4 h to remove both free and bound water from the bone matrix.^11^ Peak Force Tapping (PeakForce QNM) was applied using a high-resolution AFM system (Dimension Icon, Bruker, CA, USA) to map the surface topography (surface height), peak force error, adhesion, and DMTModulus as described previously.^54^ In brief, bone slices were scanned using RTESPA-525 probes (spring constant: 200 N/m, tip radius: 15-30 nm) with zooming in scan sizes (20 μm to 250 nm). Imaging parameters included a cantilever frequency of 2 kHz, scan rate of 0.5–1.0 Hz, and a peak force setpoint between 1 nN and 20 nN. MCFs (red arrows) and EFM (yellow arrows) regions (Fig. S4B) were visually identified from the images of surface height, peak force error, modulus, and adhesion channels (Fig. S4B). The AFM ramping mode was used to measure the in situ nanoindentation behavior of MCFs and EFM. The nanoindentation tests were conducted on the selected MCFs and EFM with a 200 nN force at a 100 nm ramping size. Force-displacement curves were recorded, processed with Bruker’s Nanoscope Analysis software, and exported for subsequent analysis. The elastic modulus was calculated using the Oliver-Pharr method after the curves were fitted with a power-law function, while the ratio (*u*_*p*_) of permanent energy dissipation was calculated from the area between the loading and unloading curves normalized to the total energy (the area below the loading curve). It should be noted that the viscoelastic behavior may affect the interpretation of AFM nanoindentation test results in PeakForce mode since the test was conducted in a dynamic mode. Nonetheless, this effect is largely negligible when testing bone samples under high loads, where the viscoelastic effect is limited.

### Picro-sirius red staining for collagen

Femurs were collected and fixed in 4% PFA for 2 days and decalcified using 10% EDTA (pH 7.5) for 3 weeks. The decalcified femurs were then embedded in paraffin, and 5-μm-thick sections were obtained. Picro-sirius red staining was performed to assess collagen fiber orientation. Briefly, paraffin-embedded sections were stained in a saturated aqueous solution of picric acid and 0.1% Direct Red 80. The sections were imaged under polarized light microscopy (Eclipse 2000, Nikon, Tokyo, Japan).

### Raman spectrometry

Raman spectrometry was conducted as previously described.^62,63^ Briefly, right femurs were thawed to room temperature prior to Raman micro-spectroscopy (InVia™ Raman microscope, Renishaw inc., Hoffman Estates, IL, USA). Raman system is equipped with 785 nm laser (Innovative Photonic Solutions, Plainsboro, Township, NJ, USA), holographic grating (1 200 lines per mm) coupled to a thermoelectrically cooled detector that gives ~1 cm^−1^ spectral resolution. Sample were placed in same orientation such that the posterior surface faced the microscope objective. Following settings were used for data acquisition: ~45 mW power, 20X objective (NA = 0.40), 10 s exposure, 5 accumulations, point scans at 5 locations at mid-diaphysis separated by ~ 300 microns. 5 spectra were averaged, and background corrected using fifth-order polynomial curve fitting to the baseline using LabSpec software (Horiba, Irvine, CA, USA). Savitzy-Golay filter was used to denoise the spectra using 4^th^ polynomial degree, window of 21. Local linear baseline was subtracted at the following wavenumbers ~740, 830, 1 145, 1 520, 1 580, and 1 720. Finally, Raman bone properties were calculated using peak intensity ratios using a custom script in Matlab r2018 (Mathworks inc., Natick, MA).

### Statistical analysis

Statistical analysis was performed using GraphPad Prism8 statistics software (GraphPad, San Diego, USA). All data are presented as mean ± SEM. One-way ANOVA was used to assess differences across three or more groups, followed by Tukey’s honestly significant difference test for pairwise comparisons when significant differences were found. T-test was used for comparing two independent groups. Assumptions for these tests included normality of data within each group, assessed using the Shapiro-Wilk test, and homogeneity of variances across groups, tested using Levene’s test. Asterisks indicate the degree of significant differences compared with the controls (*, *P* < 0.05; **, *P* < 0.01; ***, *P* < 0.001; ****, *P* < 0.000 1).

## Supplementary information

Supplementary figure S1-S4

## References

[1] Weiner, S. & Traub, W. Bone structure: from angstroms to microns. *FASEB J.* **6**, 879–885 (1992). 1740237

[2] Zimmermann, E. A. & Ritchie, R. O. Bone as a structural material. *Adv. Health Mater.* **4**, 1287–1304 (2015). 10.1002/adhm.201500070 25865873

[3] Wang, X. et al. Age-related changes in the collagen network and toughness of bone. *Bone* **31**, 1–7 (2002). 12110404 10.1016/s8756-3282(01)00697-4

[4] Burr, D. B. Bone material properties and mineral matrix contributions to fracture risk or age in women and men. *J. Musculoskelet. Neuronal. Interact.* **2**, 201–204 (2002). 15758433

[5] Wang, X. et al. The role of collagen in determining bone mechanical properties. *J. Orthop. Res*. **19**, 1021–1026 (2001). 11781000 10.1016/S0736-0266(01)00047-X

[6] Currey, J. D. Effects of differences in mineralization on the mechanical properties of bone. *Philos. Trans. R. Soc. Lond. B Biol. Sci.* **304**, 509–518 (1984). 6142490 10.1098/rstb.1984.0042

[7] Morgan, S., Poundarik, A. A. & Vashishth, D. Do non-collagenous proteins affect skeletal mechanical properties? *Calcif. Tissue Int.* **97**, 281–291 (2015). 26048282 10.1007/s00223-015-0016-3 PMC4527887

[8] Sroga, G. E. & Vashishth, D. Effects of bone matrix proteins on fracture and fragility in osteoporosis. *Curr. Osteoporos. Rep.* **10**, 141–150 (2012). 22535528 10.1007/s11914-012-0103-6 PMC3375270

[9] Lozzo, R. V. & Schaefer, L. Proteoglycan form and function: a comprehensive nomenclature of proteoglycans. *Matrix Biol.* **42**, 11–55 (2015). 25701227 10.1016/j.matbio.2015.02.003 PMC4859157

[10] Xie, C., Schaefer, L. & Iozzo, R. V. Global impact of proteoglycan science on human diseases. *iScience* **26**, 108095 (2023). 37867945 10.1016/j.isci.2023.108095 PMC10589900

[11] Nyman, J. S. et al. The influence of water removal on the strength and toughness of cortical bone. *J. Biomech.* **39**, 931–938 (2006). 16488231 10.1016/j.jbiomech.2005.01.012 PMC1941695

[12] Samuel, J. et al. Water residing in small ultrastructural spaces plays a critical role in the mechanical behavior of bone. *Bone* **59**, 199–206 (2014). 24291421 10.1016/j.bone.2013.11.018 PMC3877214

[13] Hua, R. & Jiang, J. X. Small leucine-rich proteoglycans in physiological and biomechanical function of bone. *Matrix Biol.* **11**, 100063 (2021). 10.1016/j.mbplus.2021.100063 PMC8377002 34435181

[14] Wang, X. et al. Coupling effect of water and proteoglycans on the in situ toughness of bone. *J. Bone Miner. Res.* **31**, 1026–1029 (2016). 26709950 10.1002/jbmr.2774 PMC4862903

[15] Wang, X. et al. Age-related deterioration of bone toughness is related to diminishing amount of matrix glycosaminoglycans (GAGs). *JBMR Plus* **2**, 164–173 (2018). 30009278 10.1002/jbm4.10030 PMC6042860

[16] Bianco, P. et al. Expression and localization of the two small proteoglycans biglycan and decorin in developing human skeletal and non-skeletal tissues. *J. Histochem. Cytochem.* **38**, 1549–1563 (1990). 2212616 10.1177/38.11.2212616

[17] Xu, T. et al. Targeted disruption of the biglycan gene leads to an osteoporosis-like phenotype in mice. *Nat. Genet.* **20**, 78–82 (1998). 9731537 10.1038/1746

[18] Corsi, A. et al. Phenotypic effects of biglycan deficiency are linked to collagen fibril abnormalities, are synergized by decorin deficiency, and mimic Ehlers-Danlos-like changes in bone and other connective tissues. *J. Bone Miner. Res.* **17**, 1180–1189 (2002). 12102052 10.1359/jbmr.2002.17.7.1180

[19] Lozzo, R. V. The family of the small leucine-rich proteoglycans: key regulators of matrix assembly and cellular growth. *Crit. Rev. Biochem. Mol. Biol.* **32**, 141–174 (1997). 9145286 10.3109/10409239709108551

[20] Lozzo, R. V. The biology of the small leucine-rich proteoglycans. Functional network of interactive proteins. *J. Biol. Chem.* **274**, 18843–18846 (1999). 10383378 10.1074/jbc.274.27.18843

[21] Danielson, K. G. et al. Targeted disruption of decorin leads to abnormal collagen fibril morphology and skin fragility. *J. Cell Biol.* **136**, 729–743 (1997). 9024701 10.1083/jcb.136.3.729 PMC2134287

[22] Chen, X. D. et al. The small leucine-rich proteoglycan biglycan modulates BMP-4-induced osteoblast differentiation. *FASEB J.* **18**, 948–958 (2004). 15173106 10.1096/fj.03-0899com

[23] Kram, V. et al. Biglycan in the Skeleton. *J. Histochem. Cytochem.* **68**, 747–762 (2020). 32623936 10.1369/0022155420937371 PMC7649967

[24] Hua, R. et al. Biglycan and chondroitin sulfate play pivotal roles in bone toughness via retaining bound water in bone mineral matrix. *Matrix Biol.* **94**, 95–109 (2020). 33002580 10.1016/j.matbio.2020.09.002 PMC7722036

[25] Young, M. F. et al. Small leucine-rich proteoglycans in the aging skeleton. *J. Musculoskelet. Neuronal. Interact.* **6**, 364–365 (2006). 17185826

[26] Merline, R., Schaefer, R. M. & Schaefer, L. The matricellular functions of small leucine-rich proteoglycans (SLRPs). *J. Cell Commun. Signal* **3**, 323–335 (2009). 19809894 10.1007/s12079-009-0066-2 PMC2778586

[27] Schaefer, L. & Iozzo, R. V. Biological functions of the small leucine-rich proteoglycans: from genetics to signal transduction. *J. Biol. Chem.* **283**, 21305–21309 (2008). 18463092 10.1074/jbc.R800020200 PMC2490788

[28] Wang, X. et al. Matrix protein biglycan induces osteoblast differentiation through extracellular signal-regulated kinase and Smad pathways. *Biol. Pharm. Bull.* **33**, 1891–1897 (2010). 21048317 10.1248/bpb.33.1891

[29] Lozzo, R. V. et al. Decorin is a biological ligand for the epidermal growth factor receptor. *J. Biol. Chem.* **274**, 4489–4492 (1999). 9988678 10.1074/jbc.274.8.4489

[30] Merline, R. et al. Signaling by the matrix proteoglycan decorin controls inflammation and cancer through PDCD4 and MicroRNA-21. *Sci. Signal* **4**, ra75 (2011). 22087031 10.1126/scisignal.2001868 PMC5029092

[31] Schaefer, L. et al. The matrix component biglycan is proinflammatory and signals through Toll-like receptors 4 and 2 in macrophages. *J. Clin. Invest.* **115**, 2223–2233 (2005). 16025156 10.1172/JCI23755 PMC1174916

[32] Gubbiotti, M. A. et al. Decorin interacting network: a comprehensive analysis of decorin-binding partners and their versatile functions. *Matrix Biol.* **55**, 7–21 (2016). 27693454 10.1016/j.matbio.2016.09.009 PMC6938589

[33] Waddington, R. J. et al. Differential roles for small leucine-rich proteoglycans in bone formation. *Eur. Cell Mater.* **6**, 12–21 (2003). 14562268 10.22203/ecm.v006a02

[34] Parisuthiman, D. et al. Biglycan modulates osteoblast differentiation and matrix mineralization. *J. Bone Miner. Res*. **20**, 1878–1886 (2005). 16160746 10.1359/JBMR.050612

[35] Nikitovic, D. et al. The biology of small leucine-rich proteoglycans in bone pathophysiology. *J. Biol. Chem.* **287**, 33926–33933 (2012). 22879588 10.1074/jbc.R112.379602 PMC3464503

[36] Ingram, R. T. et al. Distribution of noncollagenous proteins in the matrix of adult human bone: Evidence of anatomic and functional heterogeneity. *J. Bone Miner. Res.* **8**, 1019–1029 (1993). 8237471 10.1002/jbmr.5650080902

[37] Li, Y. et al. Age-dependent alterations of decorin glycosaminoglycans in human skin. *Sci. Rep.* **3**, 2422 (2013). 23939413 10.1038/srep02422 PMC3741628

[38] Sugars, R. V. et al. Molecular interaction of recombinant decorin and biglycan with type I collagen influences crystal growth. *Connect Tissue Res*. **44**, 189–195 (2003). 12952196 10.1080/713713596

[39] Nielsen, K. L. et al. Biglycan deficiency interferes with ovariectomy-induced bone loss. *J. Bone Miner. Res.* **18**, 2152–2158 (2003). 14672350 10.1359/jbmr.2003.18.12.2152

[40] Sims, N. A. et al. Deletion of estrogen receptors reveals a regulatory role for estrogen receptors-beta in bone remodeling in females but not in males. *Bone* **30**, 18–25 (2002). 11792560 10.1016/s8756-3282(01)00643-3

[41] Oz, O. K. et al. Bone has a sexually dimorphic response to aromatase deficiency. *J. Bone Miner. Res.* **15**, 507–514 (2000). 10750565 10.1359/jbmr.2000.15.3.507

[42] Dole, N. S. et al. TGFbeta regulation of perilacunar/canalicular remodeling is sexually dimorphic. *J. Bone Miner. Res* **35**, 1549–1561 (2020). 32282961 10.1002/jbmr.4023 PMC9126317

[43] Zhang, G. et al. Genetic evidence for the coordinated regulation of collagen fibrillogenesis in the cornea by decorin and biglycan. *J. Biol. Chem.* **284**, 8888–8897 (2009). 19136671 10.1074/jbc.M806590200 PMC2659246

[44] Han, B. et al. Differentiated activities of decorin and biglycan in the progression of post-traumatic osteoarthritis. *Osteoarthr. Cartil.* **29**, 1181–1192 (2021). 10.1016/j.joca.2021.03.019 PMC8319061 33915295

[45] Han, B. et al. Decorin regulates the aggrecan network integrity and biomechanical functions of cartilage extracellular matrix. *ACS Nano* **13**, 11320–11333 (2019). 31550133 10.1021/acsnano.9b04477 PMC6892632

[46] Li, Q. et al. Mediation of cartilage matrix degeneration and fibrillation by decorin in post-traumatic osteoarthritis. *Arthritis Rheumatol.* **72**, 1266–1277 (2020). 32162789 10.1002/art.41254 PMC7486252

[47] Robinson, K. A. et al. Decorin and biglycan are necessary for maintaining collagen fibril structure, fiber realignment, and mechanical properties of mature tendons. *Matrix Biol.* **64**, 81–93 (2017). 28882761 10.1016/j.matbio.2017.08.004 PMC5705405

[48] Samuel, J. et al. Effect of water on nanomechanics of bone is different between tension and compression. *J. Mech. Behav. Biomed. Mater.* **57**, 128–138 (2016). 26710258 10.1016/j.jmbbm.2015.12.001 PMC4798895

[49] Gandhi, N. S. & Mancera, R. L. The structure of glycosaminoglycans and their interactions with proteins. *Chem. Biol. Drug Des.* **72**, 455–482 (2008). 19090915 10.1111/j.1747-0285.2008.00741.x

[50] Bertassoni, L. E. & Swain, M. V. The contribution of proteoglycans to the mechanical behavior of mineralized tissues. *J. Mech. Behav. Biomed. Mater.* **38**, 91–104 (2014). 25043659 10.1016/j.jmbbm.2014.06.008

[51] Han, E. H. et al. Contribution of proteoglycan osmotic swelling pressure to the compressive properties of articular cartilage. *Biophys. J.* **101**, 916–924 (2011). 21843483 10.1016/j.bpj.2011.07.006 PMC3175069

[52] Unal, M., Creecy, A. & Nyman, J. S. The role of matrix composition in the mechanical behavior of bone. *Curr. Osteoporos. Rep.* **16**, 205–215 (2018). 29611037 10.1007/s11914-018-0433-0 PMC5948175

[53] Yang, P.-F. et al. Deformation regimes of collagen fibrils in cortical bone revealed by in situ morphology and elastic modulus observations under mechanical loading. *J. Mech. Behav. Biomed. Mater.* **79**, 115–121 (2018). 29291465 10.1016/j.jmbbm.2017.12.015

[54] Han, Y. et al. Removal of glycosaminoglycans affects the in situ mechanical behavior of extrafibrillar matrix in bone. *J. Mech. Behav. Biomed. Mater.* **123**, 104766 (2021). 34392037 10.1016/j.jmbbm.2021.104766 PMC8440485

[55] Wang, J. Y. & Roehrl, M. H. Glycosaminoglycans are a potential cause of rheumatoid arthritis. *Proc. Natl. Acad. Sci. USA* **99**, 14362–14367 (2002). 12391302 10.1073/pnas.222536599 PMC137889

[56] Cleland, T. P., Voegele, K. & Schweitzer, M. H. Empirical evaluation of bone extraction protocols. *PLoS One* **7**, e31443 (2012). 22348088 10.1371/journal.pone.0031443 PMC3279360

[57] Franzen, A. & Heinegard, D. Extraction and purification of proteoglycans from mature bovine bone. *Biochem. J.* **224**, 47–58 (1984). 6508769 10.1042/bj2240047 PMC1144396

[58] Wendel, M., Sommarin, Y. & Heinegard, D. Bone matrix proteins: isolation and characterization of a novel cell-binding keratan sulfate proteoglycan (osteoadherin) from bovine bone. *J. Cell Biol.* **141**, 839–847 (1998). 9566981 10.1083/jcb.141.3.839 PMC2132750

[59] Pacheco-Costa, R. et al. Connexin37 deficiency alters organic bone matrix, cortical bone geometry, and increases Wnt/beta-catenin signaling. *Bone* **97**, 105–113 (2017). 28096061 10.1016/j.bone.2017.01.010

[60] Brisby, H. et al. The effect of running exercise on intervertebral disc extracellular matrix production in a rat model. *Spine (Philos. Pa 1976)* **35**, 1429–1436 (2010). 10.1097/BRS.0b013e3181e0f5bc 20592578

[61] Ritchie, R. O. et al. Measurement of the toughness of bone: a tutorial with special reference to small animal studies. *Bone* **43**, 798–812 (2008). 18647665 10.1016/j.bone.2008.04.027 PMC3901162

[62] Bi, X. et al. Raman and mechanical properties correlate at whole bone- and tissue-levels in a genetic mouse model. *J. Biomech.* **44**, 297–303 (2011). 21035119 10.1016/j.jbiomech.2010.10.009 PMC3019269

[63] Ahmed, R. et al. Identifying bone matrix impairments in a mouse model of neurofibromatosis type 1 (NF1) by clinically translatable techniques. *J. Bone Miner. Res.* **37**, 1603–1621 (2022). 35690920 10.1002/jbmr.4633 PMC9378557

