# Supplementary material for: Pivotal roles of biglycan and decorin in regulating bone mass, water retention, and bone toughness
Source: Bone Res. 2025 Jan 2;13:2. doi: 10.1038/s41413-024-00380-2 (PMC11693767; doi:10.1038/s41413-024-00380-2)
Supplement: Supplementary file 1 — Supplementary figure S1-S4 [file 41413_2024_380_MOESM1_ESM.docx]

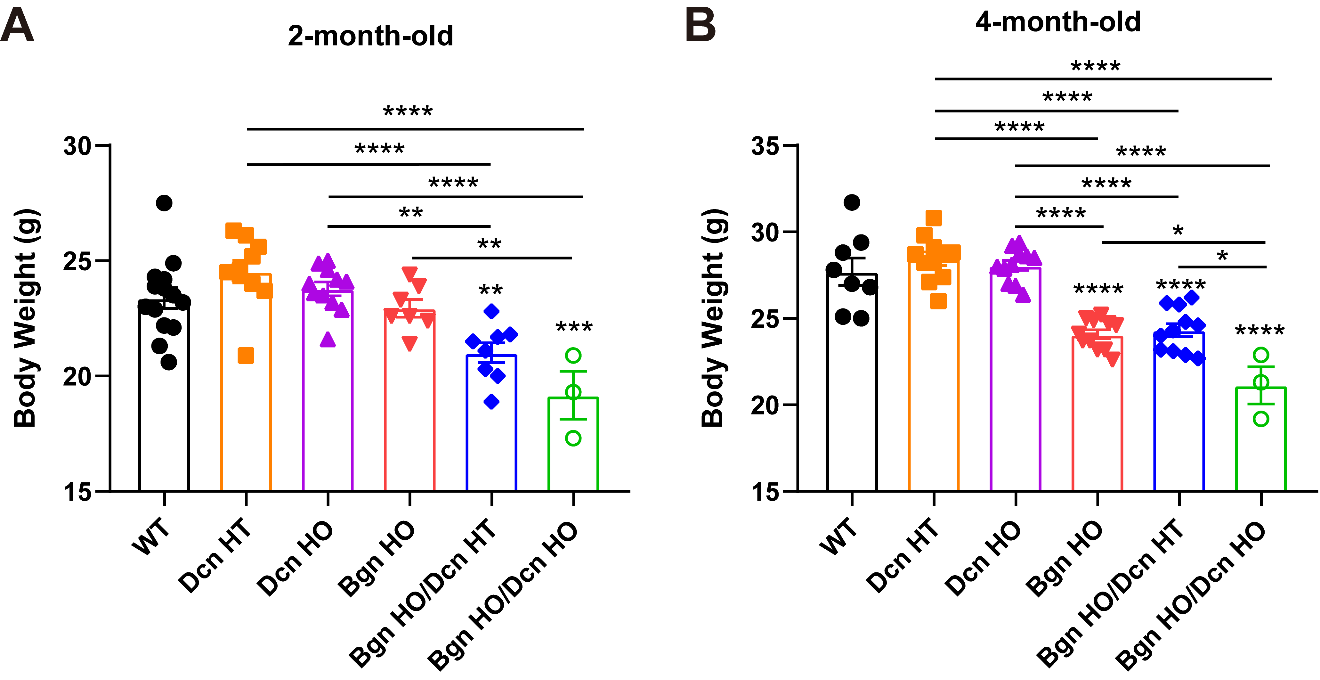


**Figure S1. *Bgn/Dcn* double KO mice showed decreased body weight during development. (A)** Summarized graphs of body weight of WT and *Bgn/Dcn* single or double KO mice at 2-month-old. WT, n=14; *Dcn* HT, n=10; *Dcn* HO, n=11; *Bgn* HO, n=7; *Bgn* HO/*Dcn* HT, n=8; *Bgn* HO/*Dcn* HO, n=3. **(B)** Summarized graphs of body weight of WT and *Bgn/Dcn* single or double KO mice at 4-month-old. WT, n=8; *Dcn* HT, n=11; *Dcn* HO, n=11; *Bgn* HO, n=11; *Bgn* HO/*Dcn* HT, n=11; *Bgn* HO/*Dcn* HO, n=3. Data are presented as mean ± SEM. One-way ANOVA with Tukey test was used for statistical analysis. *, P < 0.05; **, P < 0.01; ***, P < 0.001; ****, P < 0.0001. Asterisks above the bar graphs denote statistical comparisons with the WT groups.

**
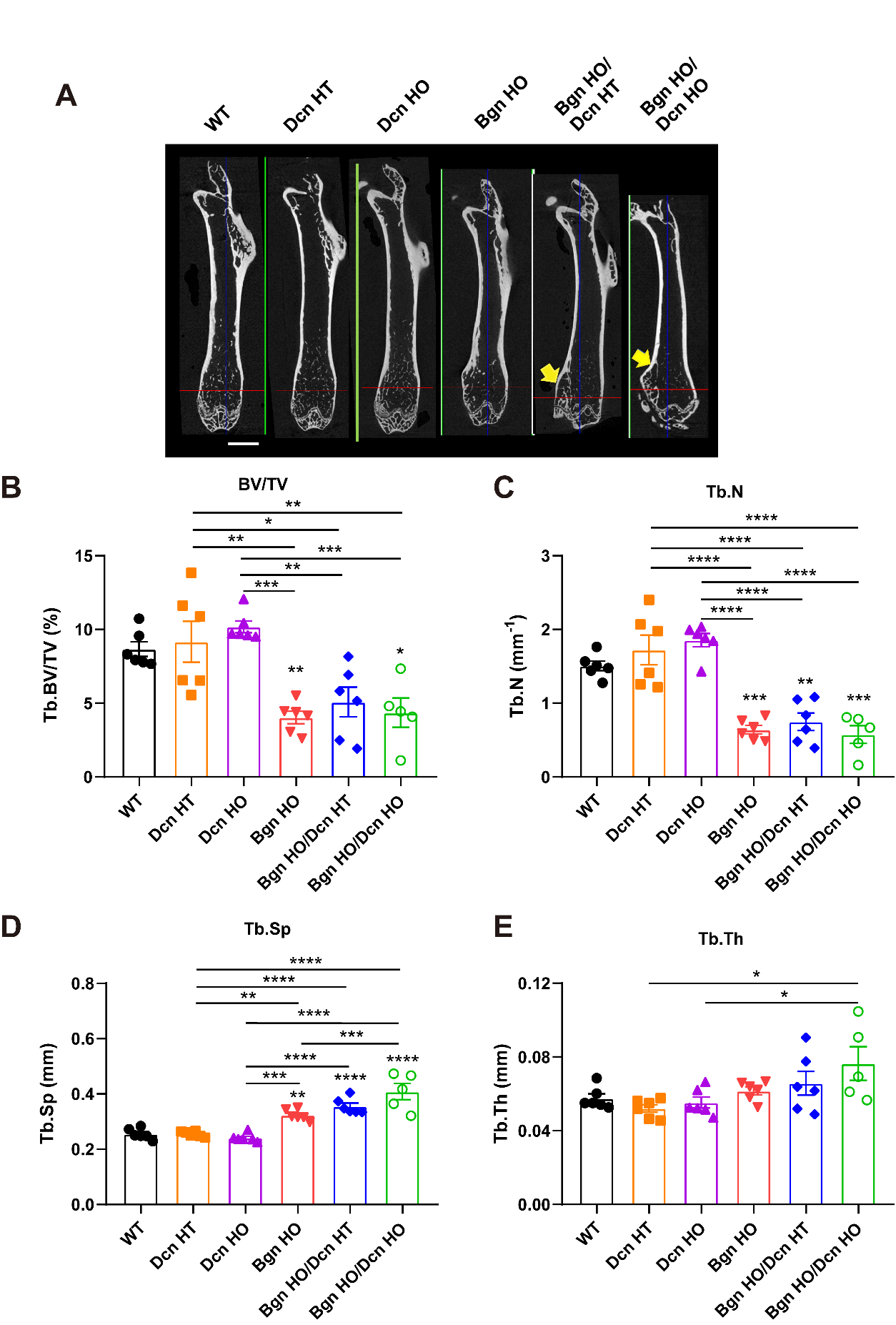
**

**Figure S2. Distal femur trabecular bone mass was reduced in *Bgn/Dcn* KO mice evaluated by microCT analysis. (A)** Representative images of the metaphyseal trabecular bone for all groups. Medial expansion in distal femur region of *Bgn/Dcn* double KO mice was indicated by yellow arrows. 3D μCT analyses of **(B)** BV/TV, **(C)** Tb.N , **(D)** Tb.Th, and **(E)** Tb.Sp of femoral trabecular bone inside the cortical shell (including the medial expansion) were performed. WT, n=6; *Dcn* HT, n=6; *Dcn* HO, n=6; *Bgn* HO, n=6; *Bgn* HO/*Dcn* HT, n=6; *Bgn* HO/*Dcn* HO, n=5. Data are presented as mean ± SEM. One-way ANOVA with Tukey test was used for statistical analysis. *, P < 0.05; **, P < 0.01; ***, P < 0.001; ****, P < 0.0001. Asterisks above the bar graphs denote statistical comparisons with the WT groups. BV, bone volume; TV, tissue volume; BV/TV, bone volume fraction; Tb.N, trabecular number; Tb.Th, trabecular thickness; Tb.Sp, trabecular separation.


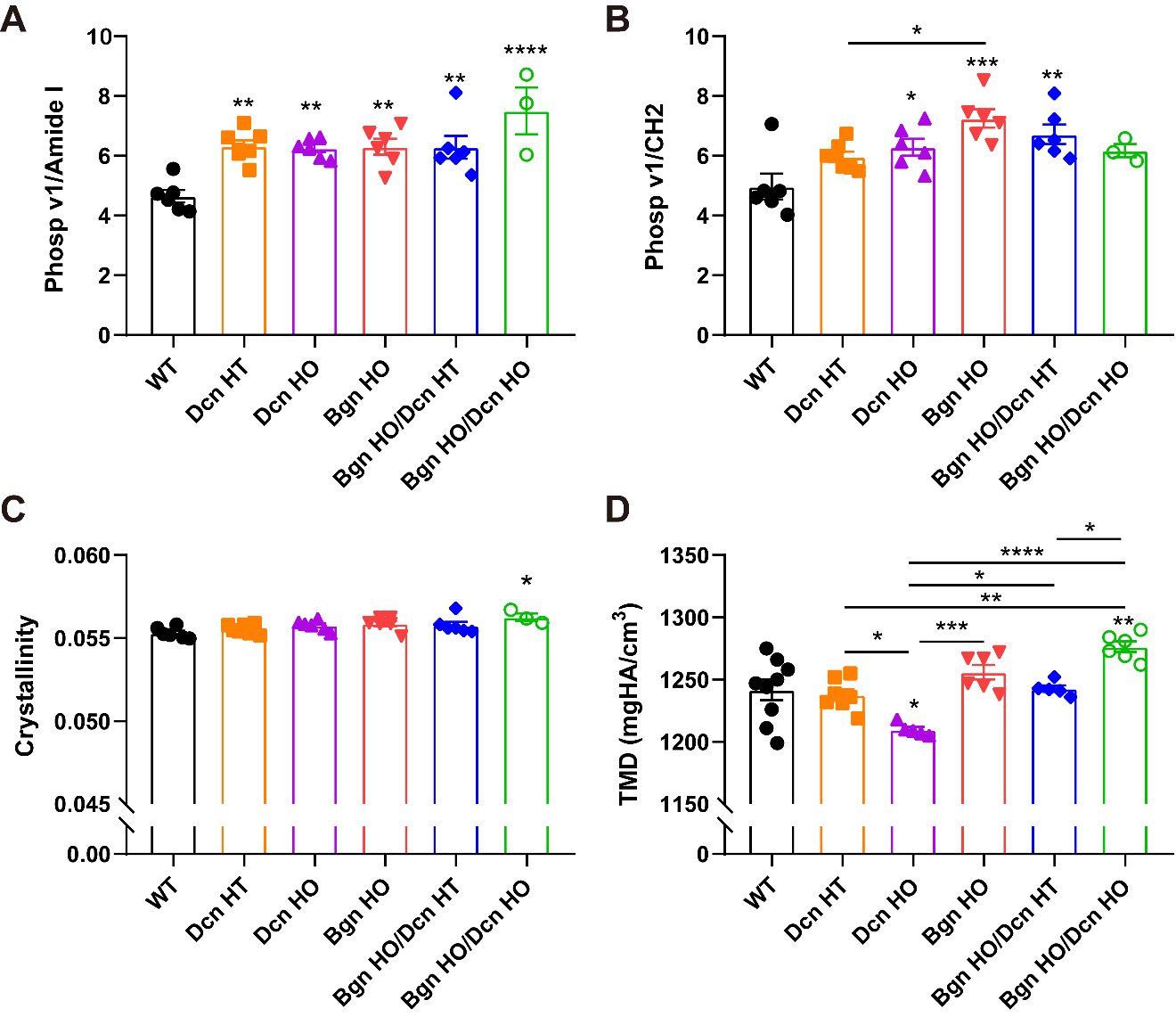


**Figure S3. Bgn/Dcn deficiency altered bone mineral matrix composition and mineral density. (A-C)** The mineral to matrix ratio and crystallinity were assessed by Raman spectroscopy in WT and *Bgn/Dcn* single or double KO mice. WT, n=6; *Dcn* HT, n=7; *Dcn* HO, n=6; *Bgn* HO, n=6; *Bgn* HO/*Dcn* HT, n=6; *Bgn* HO/*Dcn* HO, n=3. **(D)** Tissue mineral density (TMD) was determined by μCT analysis. WT, n=9; *Dcn* HT, n=7; *Dcn* HO, n=5; *Bgn* HO, n=6; *Bgn* HO/*Dcn* HT, n=5; *Bgn* HO/*Dcn* HO, n=6. Data are presented as mean ± SEM. One-way ANOVA with Tukey test was used for statistical analysis. *, P < 0.05; **, P < 0.01; ***, P < 0.001; ****, P < 0.0001. Asterisks above the bar graphs denote statistical comparisons with the WT groups.


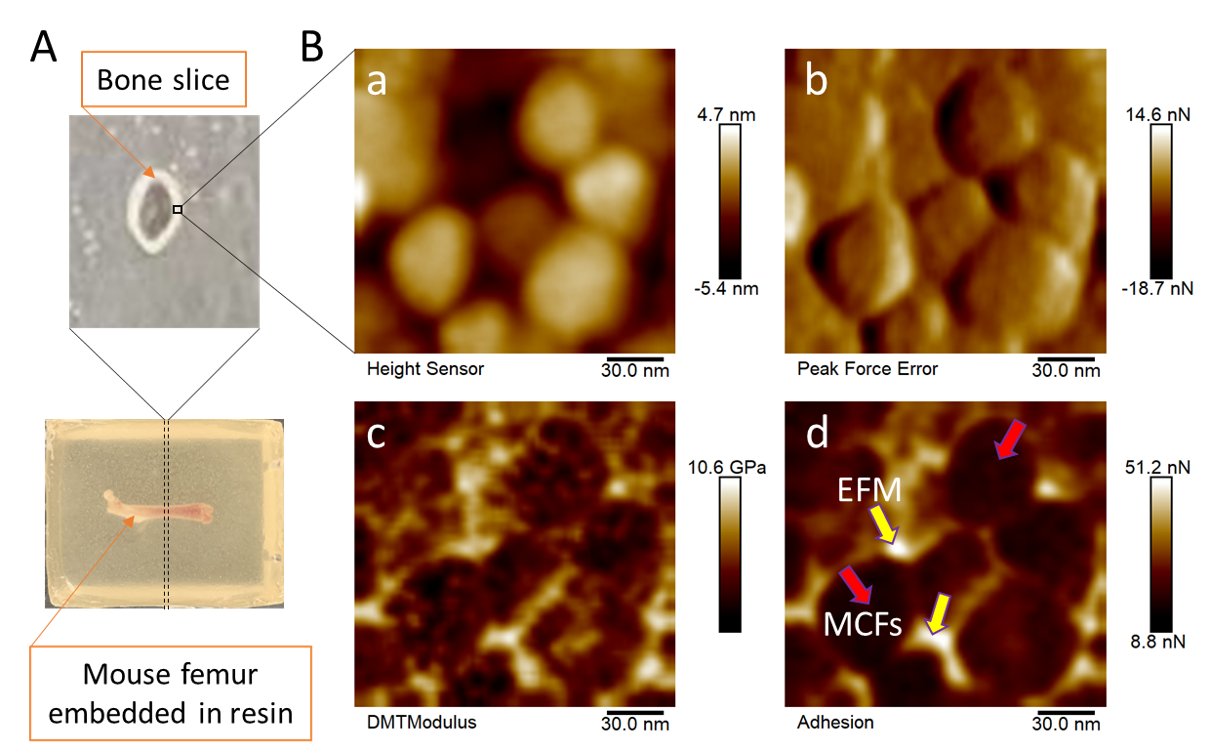


**Figure S4. Sample preparation and AFM measurements. (A)** Mouse femurs were embedded in resin and were cut from the mid-diaphysis. Bone slices were cut, ground, and polished till the thickness was about 400μm and surface roughness was less than 30 nm. **(B)** AFM image output indicating (a) surface height; (b) surface topography; (c) surface modulus mapping; and (d) surface adhesion mapping
